# Experimental phase determination with selenomethionine or mercury-derivatization in serial femtosecond crystallography

**DOI:** 10.1107/S2052252517008557

**Published:** 2017-08-08

**Authors:** Keitaro Yamashita, Naoyuki Kuwabara, Takanori Nakane, Tomohiro Murai, Eiichi Mizohata, Michihiro Sugahara, Dongqing Pan, Tetsuya Masuda, Mamoru Suzuki, Tomomi Sato, Atsushi Kodan, Tomohiro Yamaguchi, Eriko Nango, Tomoyuki Tanaka, Kensuke Tono, Yasumasa Joti, Takashi Kameshima, Takaki Hatsui, Makina Yabashi, Hiroshi Manya, Tamao Endo, Ryuichi Kato, Toshiya Senda, Hiroaki Kato, So Iwata, Hideo Ago, Masaki Yamamoto, Fumiaki Yumoto, Toru Nakatsu

**Affiliations:** a RIKEN SPring-8 Center, 1-1-1 Kouto, Sayo-cho, Sayo-gun, Hyogo 679-5148, Japan; b Structural Biology Research Center, Photon Factory, Institute of Materials Structure Science, KEK/High Energy Accelerator Research Organization, 1-1 Oho, Tsukuba, Ibaraki 305-0801, Japan; c Department of Biological Sciences, Graduate School of Science, The University of Tokyo, 7-3-1 Hongo, Bunkyo-ku, Tokyo 113-0033, Japan; d Department of Structural Biology, Graduate School of Pharmaceutical Sciences, Kyoto University, 46-29 Yoshida-Shimoadachi-cho, Sakyo-ku, Kyoto 606-8501, Japan; e Department of Applied Chemistry, Graduate School of Engineering, Osaka University, 2-1 Yamadaoka, Suita, Osaka 565-0871, Japan; f Division of Food Science and Biotechnology, Graduate School of Agriculture, Kyoto University, Gokasho, Uji, Kyoto 611-0011, Japan; g Research Center for Structural and Functional Proteomics, Institute for Protein Research, Osaka University, 3-2 Yamadaoka, Suita, Osaka 565-0871, Japan; h Institute for Integrated Cell-Material Sciences, Kyoto University, Yoshida Ushinomiya-cho, Sakyo-ku, Kyoto 606-8501, Japan; i Japan Synchrotron Radiation Research Institute, 1-1-1 Kouto, Sayo-cho, Sayo-gun, Hyogo 679-5198, Japan; j Molecular Glycobiology, Research Team for Mechanism of Aging, Tokyo Metropolitan Geriatric Hospital and Institute of Gerontology, 35-2 Sakae-cho, Itabashi-ku, Tokyo 173-0015, Japan; k Department of Cell Biology, Graduate School of Medicine, Kyoto University, Yoshidakonoe-cho, Sakyo-ku, Kyoto 606-8501, Japan

**Keywords:** serial femtosecond crystallography, SAD phasing, XFELs, selenomethionine derivatization, mercury soaking

## Abstract

Serial femtosecond crystallography (SFX) using X-ray free-electron lasers (XFELs) holds enormous potential for the structure determination of proteins for which it is difficult to produce large and high-quality crystals. SFX has been applied to various systems, but rarely to proteins that have previously unknown structures. Consequently, the majority of previously obtained SFX structures have been solved by the molecular replacement method. To facilitate protein structure determination by SFX, it is essential to establish phasing methods that work efficiently for SFX. Here, selenomethionine derivatization and mercury soaking have been investigated for SFX experiments using the high-energy XFEL at the SPring-8 Angstrom Compact Free-Electron Laser (SACLA), Hyogo, Japan. Three successful cases are reported of single-wavelength anomalous diffraction (SAD) phasing using X-rays of less than 1 Å wavelength with reasonable numbers of diffraction patterns (13 000, 60 000 and 11 000). It is demonstrated that the combination of high-energy X-rays from an XFEL and commonly used heavy-atom incorporation techniques will enable routine *de novo* structural determination of biomacromolecules.

## Abstract 2

High-energy X-rays are essential for *de novo* structure determination with strong anomalous scattering from selenium or mercury. Single-wavelength anomalous diffraction phasing using selenomethionine-derivatization and mercury-soaking techniques has been successfully applied to serial femtosecond crystallography with 13.0 keV or 12.6 keV X-rays produced at SACLA.

## 1. Introduction   

X-ray free-electron lasers (XFELs) offer many new opportunities in protein crystallography (Schlichting, 2015 ▸; Neutze *et al.*, 2015 ▸). Serial femtosecond crystallography (SFX) has been used to elucidate molecular structures from micron-sized protein crystals at ambient temperatures (Chapman *et al.*, 2011 ▸; Redecke *et al.*, 2013 ▸; Kupitz *et al.*, 2014 ▸; Tenboer *et al.*, 2014 ▸; Kang *et al.*, 2015 ▸). In addition to the regular application of this approach to a variety of proteins and protein complexes, considerable efforts have been focused on *de novo* structure determination methods (Spence *et al.*, 2011 ▸; Son *et al.*, 2011 ▸; Barends *et al.*, 2014 ▸). In order to determine crystal structures using microcrystals that are unsuitable for conventional synchrotron radiation (SR) beamlines, it is essential to develop effective experimental phasing methods in SFX.

Phase determination is a central problem in protein crystallography and single-wavelength anomalous diffraction (SAD) (Wang, 1985 ▸) is the most commonly used experimental phasing method (Rose & Wang, 2016 ▸). The successful application of SAD techniques to macromolecular crystallography with SR had led to these techniques becoming more widely used with SFX. Barends and co-workers used the SAD method to achieve *de novo* phasing of SFX data from tetragonal lysozyme crystals with 8.5 keV X-rays at the LCLS (Linac Coherent Light Source, California, USA) (Barends *et al.*, 2014 ▸). They used ∼60 000 single-pulse diffraction patterns of crystals, prepared with gadolinium derivatives, and obtained an automatically traceable electron-density map. The number of patterns required was recently reduced to 7 000 by improved data processing (Nass *et al.*, 2016 ▸). Nakane and co-workers succeeded in SAD phasing of lysozyme using anomalous signals from intrinsic sulfur atoms and a bound chloride ion with the 7 keV beam at SACLA (SPring-8 Angstrom Compact Free-Electron Laser, Hyogo, Japan) (Nakane *et al.*, 2015 ▸). Nass and co-workers and Batyuk and co-workers also reported sulfur-SAD phasing of thaumatin and human A_2A_ adenosine receptor, respectively, at 6 keV at the LCLS (Nass *et al.*, 2016 ▸; Batyuk *et al.*, 2016 ▸). Fukuda and co-workers determined the structure of *Alcaligenes faecalis* nitrite reductase by the SAD method using intrinsically bound copper ions (Fukuda, Tse, Nakane *et al.*, 2016 ▸). Nakane and co-workers demonstrated that iodine-labeled detergent can be used for SAD, SIR (single isomorphous replacement) and SIRAS (SIR with anomalous scattering) phasing of membrane proteins (Nakane, Hanashima *et al.*, 2016 ▸). Colletier and co-workers successfully determined the structure of the BinAB toxin from nanocrystals using iodine, gadolinium and mercury atoms with the MIRAS (multiple isomorphous replacement with anomalous scattering) method (Colletier *et al.*, 2016 ▸).

Heavy atoms such as selenium, mercury, gold and platinum, which have absorption edges at 12.658, 12.284, 11.919 and 11.564 keV, respectively, are frequently used to acquire phases with SR. Among these elements, mercury and selenium are the most commonly used in phasing. Mercury was first applied to the structure determination of hemoglobin by pioneers in the protein crystallography field (Perutz *et al.*, 1960 ▸). Selenium can be incorporated into recombinant proteins as selenomethionine (SeMet) derivatives (Hendrickson *et al.*, 1990 ▸). Methods for preparing Hg- or Se-containing crystals have been established and applied to many cases in protein crystallography, so the methods can also be easily applied to SFX provided that sufficiently high-energy X-rays are available. Moreover, high-energy X-rays enable high-resolution data collection with fixed detector dimensions. SACLA was designed to produce femtosecond X-ray pulses for these purposes and at even higher photon energies (Ishikawa *et al.*, 2012 ▸).

Recently, Se-SAD phasing at the LCLS using a selenobiotin-bound protein was reported (Hunter *et al.*, 2016 ▸). In that study a large number (481 079) of patterns was required owing to the small fraction of Se incorporation. Here, we report two successful cases of SAD phasing of SeMet-labeled proteins with SFX data by taking advantage of the high-energy (13 keV) X-rays available at SACLA. Only 13 000 patterns were necessary to solve the structure at 1.4 Å in an orthorhombic space group with a Bijvoet ratio 〈|Δ*F*_ano_|〉/〈|*F*|〉 of 3.7%. Another case of a hexagonal space group having an indexing ambiguity with a Bijvoet ratio of 2.2% required 60 000 patterns at 1.5 Å. Moreover, improvements in spot prediction accuracy and intensity scaling enabled SAD phasing of Hg-bound luciferin-regenerating enzyme (LRE), which has not previously been solved by SAD (Yamashita *et al.*, 2015 ▸). Determination of this structure required 11 000 patterns at 1.5 Å resolution.

## 2. Materials and methods   

### 2.1. Purification of Se-Met Stem and ACG   

The stem domain of human POMGnT1 (92–250) (UniProt ID Q8WZA1) was subcloned into pGEX-6P-1 (GE Healthcare) and expressed in *Escherichia coli* B834(DE3) (Merck Millipore) in the presence of 0.1 mg l^−1^ ampicillin (Wako). Construction of ACG (*Agrocybe cylindracea* galectin) fused with a FLAG tag at its N-terminus was performed as described previously (Hu *et al.*, 2013 ▸) and expressed in *E. coli* B834(DE3) in the presence of 0.05 mg l^−1^ kanamycin (Wako). The transformed cells were cultured in LeMaster medium (Nihon Pharmaceutical) supplemented with 5 mg ml^−1^ l-Se-methionine, 1% glucose, and KAO and MICHAYLUK vitamin solutions (Sigma–Aldrich). Protein expression was induced by the addition of 0.5 m*M* isopropyl β-d-1-thio­galacto­pyranoside (IPTG) (Wako) at OD_600_ = 0.5–0.7, and the cells were further incubated overnight at 16°C. The harvested cells were disrupted by sonication (Tomy Seiko) and the insoluble fraction was removed by centrifugation. The recombinant stem domain was purified by glutathione-Sepharose 4B affinity chromatography (GE Healthcare) and the GST tag was removed with PreScission protease (GE Healthcare) on the column. The sample was passed through Q Sepharose (GE Healthcare) and loaded onto a Superdex75 10/300GL (GE Healthcare) column equilibrated with 10 m*M* HEPES–NaOH (pH 7.0), 100 m*M* NaCl and 1 m*M* DTT. The recombinant ACG was purified by α-lactose-agarose affinity chromatography (Sigma–Aldrich). ACG protein was eluted by 0.2 *M* lactose from the column. The eluent was diluted by 0.1 *M* HEPES–NaOH (pH 7.5) and passed through Q Sepharose, then loaded onto a Superdex200 10/300GL (GE Healthcare) column equilibrated with 10 m*M* HEPES–NaOH (pH 7.0), 100 m*M* NaCl and 1 m*M* DTT. The purified samples were concentrated to 15–30 mg ml^−1^, frozen in liquid nitrogen and stored at −80°C.

### 2.2. Crystallization of Se-Met Stem and ACG   

Large crystals of Stem with 4-nitrophenyl β-d-manno­pyranoside and ACG with blood type A tetraose type 2 (ELICITYL) were obtained by the hanging-drop vapor diffusion method at 20°C. The reservoir solution conditions and the crystal structures have been described previously (Kuwabara *et al.*, 2013 ▸, 2016 ▸). Briefly, the reservoir solution condition for Stem was 0.1 *M* HEPES–NaOH (pH 7.0) and 16–18% PEG 4000. The conditions for ACG were 26–32% PEG 1500.

Crystals with a diameter of 200–300 µm appeared within one week. Microcrystals for SFX were prepared by the rotational seeding crystallization technique (Fukuda, Tse, Suzuki *et al.*, 2016 ▸) as follows. A few large crystals were suspended in 20 µl of reservoir solution and the mixtures were diluted up to 600 µl. The sample was sonicated and centrifuged for 2–3 s at 2000*g*, and the supernatant was recovered as a seed solution. Before crystallization, 5 m*M* 4-nitrophenyl β-d-manno­pyranoside and 2.5 m*M* blood type A tetraose type 2 were mixed with Stem and ACG, respectively. In a 0.7 ml tube, 100 µl of 10–15 mg ml^−1^ protein solution was mixed with 100 µl of 16–18% PEG 4000 (for Stem) or 32% PEG 1500 (for ACG), and then 2 µl of the seed solution was added. The tube was rotated on an RT-50 rotator at 50 rpm for 1–2 d at 20°C. The microcrystal solution was filtered through a 30 µm CellTrics filter (Chiyoda Science) and adjusted to a number density of approximately 2–6 × 10^6^ crystals ml^−1^.

### 2.3. Crystallization of LRE-Hg   

Preparation of LRE-Hg crystals was performed as described previously (Yamashita *et al.*, 2015 ▸). Briefly, rod-shaped crystals of sizes 2–5 × 10–30 µm were obtained with reservoir solution conditions of 35% PEG3350, 10% MPD, 0.1 *M* HEPES pH 7.5, 0.2 *M* MgCl_2_ by the batch method with micro seeding. The native crystals were soaked in the stock solution containing 1 m*M* HgO for 6 d and then back-soaked in the stock solution for 1 h to obtain Hg-derivative crystals. The stock solution condition was 31.3% PEG 3350, 0.1 *M* HEPES (pH 7.5), 10% MPD, 0.2 *M* MgCl_2_, 0.1 *M* NaCl and 5% glycerol.

### 2.4. XFEL experiments   

The microcrystal suspension was concentrated by centrifugation for 5–10 s at 2000*g*. The sample was mixed with a grease matrix and packed into an injector syringe (No. 7803-05, Hamilton) before data collection (Sugahara *et al.*, 2015 ▸). The syringe injector system was installed on a DAPHNIS (Diverse Application Platform for Hard X-ray Diffraction in SACLA) chamber (Tono *et al.*, 2015 ▸) in a moist helium environment at room temperature. The beam was focused with KB mirrors (Yumoto *et al.*, 2013 ▸). The pulse duration was <10 fs and the repetition rate was 30 Hz. The diffraction patterns were collected with XFEL radiation on BL3 (EH4) of SACLA (Hyogo, Japan) using an MPCCD detector with a short working distance (SWD) octal sensor arrangement (Kameshima *et al.*, 2014 ▸). The grease type, crystal size, filter pore size, nozzle aperture, flow rate, crystal density in the stream, beam size, photon energy and detector distance for each sample are summarized in Table S1 in the supporting information. The total amounts of sample used were 5, 30 and 3+10 mg for Stem, ACG and LRE (original+additional), respectively. The total data collection times were approximately 1, 5.5 and 2.5+17 h, respectively. The anomalous scattering contributions 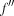 of Se and Hg at photon energies of 13.0 and 12.6 keV were 3.64 e and 9.75 e, respectively. The Bijvoet ratio was calculated using the approximate equation (Hendrickson & Teeter, 1981 ▸).

### 2.5. SFX data processing   

Prior to data processing with *CrystFEL*, images were filtered through *Cheetah* (Barty *et al.*, 2014 ▸) adapted for the SACLA API (Joti *et al.*, 2015 ▸). Images with more than 20 diffraction spots were retained as ‘hits’. We used the *CrystFEL* software suite (Version 0.6.1; White *et al.*, 2012 ▸) for peak search, indexing, integration and merging. In peak search, the related parameters minimum threshold, minimum gradient and minimum SNR (signal-to-noise ratio) were optimized to maximize the number of indexed patterns. The values of these parameters were 500, 200 000, 1 (Stem), 400, 10 000, 5 (ACG) and 200, 50 000, 1 (LRE). The detector distance was determined with a grid search by maximizing the number of indexed patterns. Indexing was performed using *DIRAX* (Version 1.16; Duisenberg, 1992 ▸) and *MOSFLM* (Version 7.2.0; Leslie & Powell, 2007 ▸) in that order, and the first successful indexing result was used for integration. The Bragg intensity integration was performed with direct summation with three concentric rings (default). The radii of the rings were 4, 6 and 8. In integration, no pixel value cutoff was employed and overloaded reflections were integrated. The distributions of detector pixel values, background and Bragg intensity values are shown in Figs. S1 and S2 in the supporting information. The median values of the unit-cell parameters were used in downstream analyses. A histogram of unit-cell parameters determined for each pattern is shown in Fig. S3 in the supporting information.

We used a low-angle X-ray absorber for LRE-Hg data collection. To correct the intensities in the low-resolution area, the absorber radius and center position were determined by inspecting the diffraction images. Before Monte Carlo integration, the integrated intensities and measurement errors of the spots in the absorber region were corrected by the theoretical transmission factor. The angular dependence was not taken into account here.

Integrated intensities on each pattern were merged using *partialator* in the *CrystFEL* suite with the partiality model of *unity*. *CrystFEL* determined the high-resolution limit for each pattern based on peak search results (White *et al.*, 2016 ▸). When merging, the high-resolution limit (*d* ^−1^) in each pattern was extended by 1.8 nm^−1^ (Stem-Se), 1.5 nm^−1^ (ACG-Se) or 1.2 nm^−1^ (LRE-Hg) using the --push-res option. In the test of the number of patterns required for SAD phasing, the first patterns in the list were successively merged without any reordering or selection of patterns.

The SACLA High-Performance Computing (HPC) system was used for computation. Up to 16 nodes were used simultaneously, each of which consisted of two Intel Xeon X5690 processors operating at 3.47 GHz (12 threads) and 24 GB memory, and it took 1–6 h to process the data for each case.

### 2.6. SAD phasing and refinement   

The initial phases were determined and improved using *SHELXC* (Version 2013/2), *SHELXD* (Version 2013/1) and *SHELXE* (Version 2016/1) with the auto-tracing feature (Sheldrick, 2010 ▸). In the case of Stem and ACG, the asymmetric unit was assumed to contain two molecules with solvent contents of 54% and 63.5%, respectively. In the case of LRE, the asymmetric unit was assumed to contain one molecule with a solvent content of 44%. The high-resolution cutoff values in the substructure search by *SHELXD* were 1.9 (Stem-Se), 2.0 (ACG-Se) and 2.1 Å (LRE-Hg), which were decided by *SHELXC*. Substructure optimization was not performed in *SHELXE*. Initial model building with iterative refinement by *REFMAC* (Version 5.8.0049; Murshudov *et al.*, 2011 ▸) was performed using *Buccaneer* (Version 1.5.2; Cowtan, 2006 ▸). Two CPU cores of a node of the SACLA HPC system were used for computation and it took a few hours for these procedures. Manual model rebuilding with *Coot* (Version 0.8-pre; Emsley *et al.*, 2010 ▸) and refinement using *phenix.refine* (Version 1.9; Afonine *et al.*, 2012 ▸) were repeated. The stereochemical properties of the final refined model were analyzed with *PHENIX* (Adams *et al.*, 2010 ▸) including *MolProbity* analysis (Chen *et al.*, 2010 ▸). Tabulated values of atomic form factors, 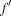 and 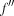, were used for the selenium and mercury atoms (Sasaki, 1989 ▸). All molecular graphics figures were prepared using *PyMOL* (Version 1.3r1; Schrödinger LLC).

## 3. Results and discussion   

### 3.1. SAD phasing with SeMet-labeled Stem crystals   

SeMet-labeled Stem (the stem domain of human POMGnT1) (Kuwabara *et al.*, 2016 ▸) crystallizes in the space group *P*2_1_2_1_2. Diffraction data from SeMet-Stem were collected at a wavelength of 0.954 Å (13.0 keV). There are two monomers in the asymmetric unit and each polypeptide consists of 164 amino acid residues, of which three methionine residues were replaced with SeMet (Bijvoet ratio ≃ 3.7%). Out of 91 437 collected images, 35 295 (38.6%) were selected using the *Cheetah* pipeline adapted for SACLA (Nakane, Joti *et al.*, 2016 ▸). Of these images, 26 583 (75.3%) patterns were indexed and integrated using the *CrystFEL* suite, with sensor geometry refined by *geoptimiser* (Yefanov *et al.*, 2015 ▸). The integrated intensities of all the diffraction patterns were scaled and merged with *partialator* in the *CrystFEL* suite using the partiality model of *unity*, which refined the linear scale and *B* factor for each crystal and merged them without partiality corrections.

**Figure 1:**
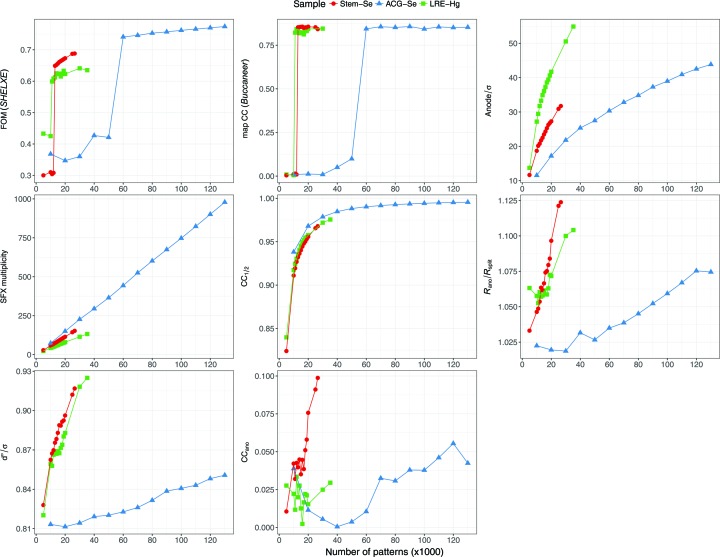
Data quality and phasing statistics as a function of the number of patterns. FOM is reported by *SHELXE* for the correct hand. Map CC is the real-space CC between the model built by *Buccaneer* and the final refined 2*mF*_o_ − *DF*_c_ map. ‘Anode’ is the maximum peak height of the anomalous difference Fourier map calculated by *ANODE* (Thorn & Sheldrick, 2011 ▸) with the refined model. 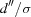 was calculated with *F_A_* and σ(*F_A_*) in the output of *SHELXC* (Sheldrick, 2010 ▸). The high-resolution cutoffs for Stem-Se, ACG-Se and LRE-Hg are 1.4, 1.5 and 1.5 Å, respectively. Note that the reason why the overall multiplicities do not increase in the same way despite the same Laue symmetry (for Stem-Se and LRE-Hg) is (i) a different resolution cutoff, (ii) a per-pattern resolution cutoff in merging, and (iii) different reciprocal-lattice point sizes determined for each pattern. This figure was prepared using *ggplot2* (Wickham, 2009 ▸) in *R* (R Development Core Team, 2008 ▸).

We tested various numbers of patterns in SAD phasing using a custom-made script. The script first ran *partialator* with the given number of patterns, and then attempted SAD phasing using the *SHELXC*, *D* and *E* programs. Heavy-atom sites determined by *SHELXD* were passed to *SHELXE*, which performed phasing and phase improvement including polyalanine chain tracing. As chain tracing was performed, there was a sharp rise in the figure of merit (FOM) when the correct solution was attained (Fig. 1 ▸). At least 13 000 patterns (a mean multiplicity of 74.3 at 1.4 Å resolution) were required for SAD phase determination. The localization of Se sites required 13 000 patterns (Fig. S4 in the supporting information). Data collection, phasing and refinement statistics for the minimum set of patterns and for all patterns are summarized in Table S2 in the supporting information.

**Figure 2:**
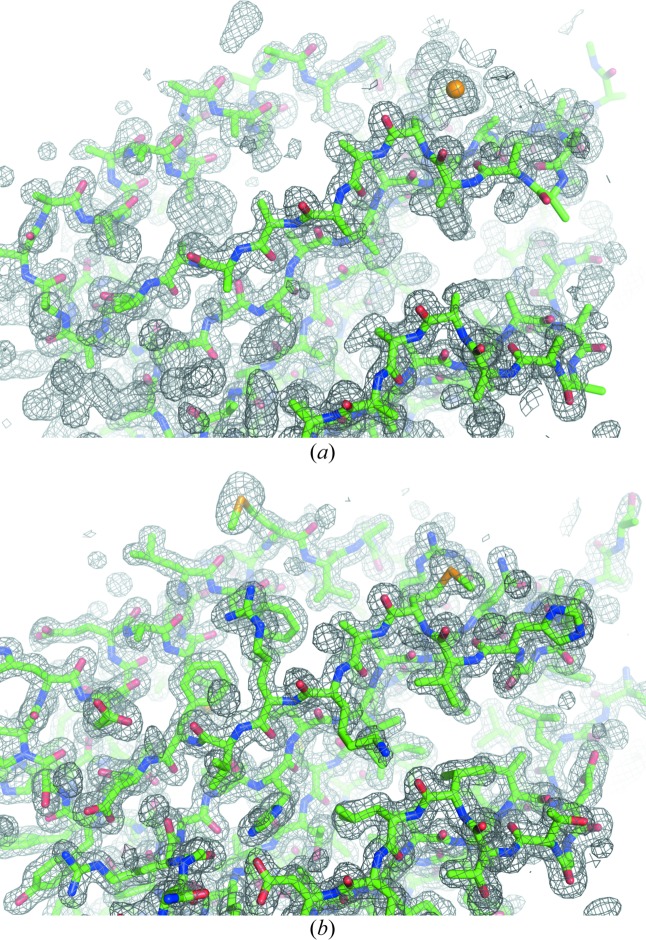
Initial and final maps and models of Se-Met Stem. (*a*) An experimentally phased map and traced polyalanine model. (*b*) A 2*mF*_o_ − *DF*_c_ map and refined model. 13 000 indexed patterns of SeMet-derivative crystals were used for the calculation. Electron-density maps are contoured at 1.0σ.

From 13 000 patterns, we identified the positions of three Se atoms in the asymmetric unit using *SHELXD* as CC_all_ of 9.21% and CC_weak_ of 5.42%. The CC values were weighted by the estimated standard deviation of observations, which probably contributed to their small values (for all patterns, CC_all_ and CC_weak_ were 18.55% and 14.19%, respectively; see Fig. S5 in the supporting information). When the correct hand was used in *SHELXE*, a mean FOM of 0.649 was obtained, and 285 residues were modeled with CC = 42.5%. The electron-density map for the correct hand was readily interpretable (Fig. 2 ▸). Automatic model building was then performed using *Buccaneer* with *REFMAC5* (Murshudov *et al.*, 2011 ▸). A total of 300 residues (out of 306) were modeled with satisfactory accuracy (*R*_work_ = 24.20% and *R*_free_ = 31.63%). After a few cycles of manual model rebuilding using *Coot* and automated refinement using *phenix.refine*, the refinement converged with residuals *R*_work_ = 17.04% and *R*_free_ = 20.67%. In the anomalous difference Fourier map, six Se sites with peak heights of 20.6σ, 19.4σ, 18.0σ, 18.0σ, 11.2σ and 6.1σ were identified (Fig. S6*a* in the supporting information). Anomalous difference Patterson maps of 13 000 and 26 583 patterns are shown in Fig. S7 in the supporting information.

### 3.2. SAD phasing with SeMet-labeled ACG crystals (a case of indexing ambiguity)   

SeMet-labeled ACG (*Agrocybe cylindracea* galectin) (Kuwabara *et al.*, 2013 ▸) crystals belong to the space group *P*6_5_ and have two monomers in the asymmetric unit. Each polypeptide consists of 178 residues and contains two Met residues; however, the first set of residues, including one Met residue, is disordered and the Bijvoet ratio is ∼2.2% at 13.0 keV. As the lattice symmetry (6/*mmm*) is higher than the Laue symmetry (6/*m*), there was a need to resolve the indexing ambiguity. The lower Bijvoet ratio and indexing ambiguity complicate the structure determination.

The data processing scheme was the same as that described above, with the exception of resolving the indexing ambiguity. Prior to merging using *partialator*, the twofold indexing ambiguity (*hkl* and 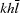) was resolved using *ambigator* in the *CrystFEL* suite. The numbers of collected, hit or indexed images are summarized in Table 1 ▸.

**Figure 3:**
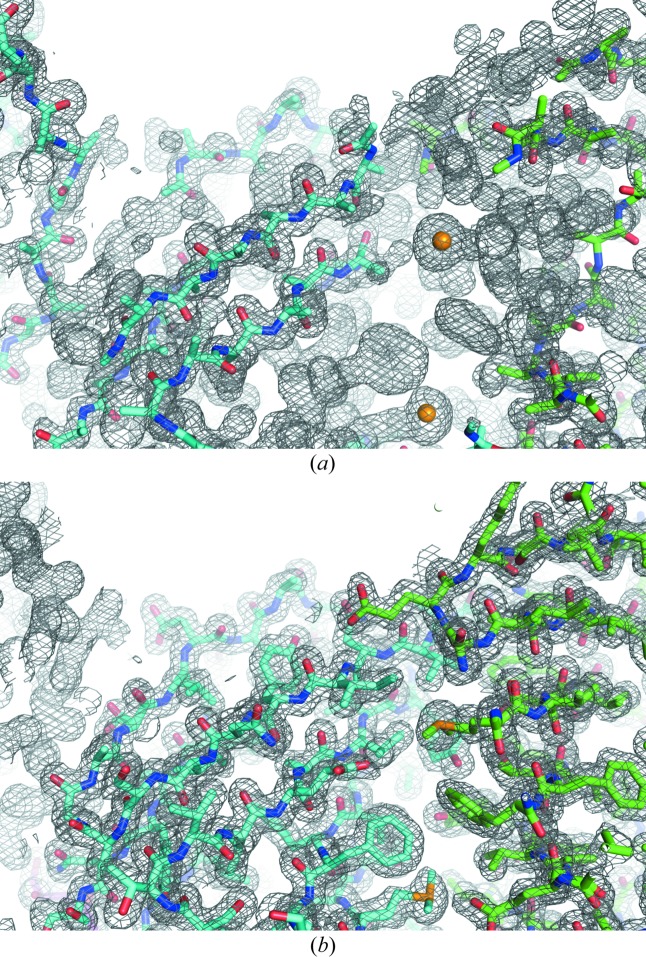
Initial and final maps and models of ACG. (*a*) An experimentally phased map and traced polyalanine model. (*b*) A 2*mF*_o_ − *DF*_c_ map and refined model. 60 000 indexed patterns of SeMet-ACG crystals were used for the calculation. Electron-density maps are contoured at 1.0σ.

We tested various numbers of patterns in the SAD phasing in the same way. At least 60 000 patterns (a mean multiplicity of 443.4 at 1.5 Å resolution) were required for SAD phase determination (Fig. 1 ▸). The localization of Se sites required 35 000 patterns (Fig. S4 in the supporting information). The electron-density map from 60 000 patterns was readily interpretable (Fig. 3 ▸). In the anomalous difference Fourier map, two Se sites with peak heights of 24.6σ and 24.1σ were identified (Fig. S6*b* in the supporting information). The correctness of the indexing ambiguity resolution was confirmed by comparison with the refined model (Fig. S8 in the supporting information). Data collection, phasing and refinement statistics for the minimum set of patterns and for all patterns are summarized in Table S3 in the supporting information.

The number of patterns required for the ACG-Se case was much larger than others. This could be due to the lower fraction of anomalous scatterers. The actual Bijvoet ratio of ACG-Se calculated using *F*_model_ of the refined structure (1.4%) was three times lower than that of Stem-Se (Table 1 ▸), which would require nine times larger multiplicity to achieve the same level of anomalous signal-to-noise ratio. As the Laue symmetry of ACG-Se is 1.5 times higher than the other cases, the required number of patterns would be about six times the others’, which is close to the actual value.

### 3.3. SAD phasing with Hg-soaked LRE crystals   

In our earlier study (Yamashita *et al.*, 2015 ▸) using *CrystFEL* Version 0.5.3a, we found that SAD phasing of Hg-derivative microcrystals of LRE (Gomi & Kajiyama, 2001 ▸) at a wavelength of 0.984 Å (12.6 keV) was not successful. Because data processing methods have improved significantly since that time, we reprocessed the raw data using state-of-the-art methods. The numbers of collected, hit or indexed images are summarized in Table 1 ▸. The number of indexed images was comparable with our previous result (34 393 patterns).

The intensities were merged in the same way using *partialator*, where four patterns with relative *B* factors that were too high (|*B*| 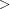 100 Å^2^) were discarded. Prediction refinement and the use of *partialator* in *CrystFEL* Version 0.6.1, which were not available in the previously used *CrystFEL* (Version 0.5.3a), considerably improved the data quality over the entire resolution range (Fig. S9 in the supporting information).

**Figure 4:**
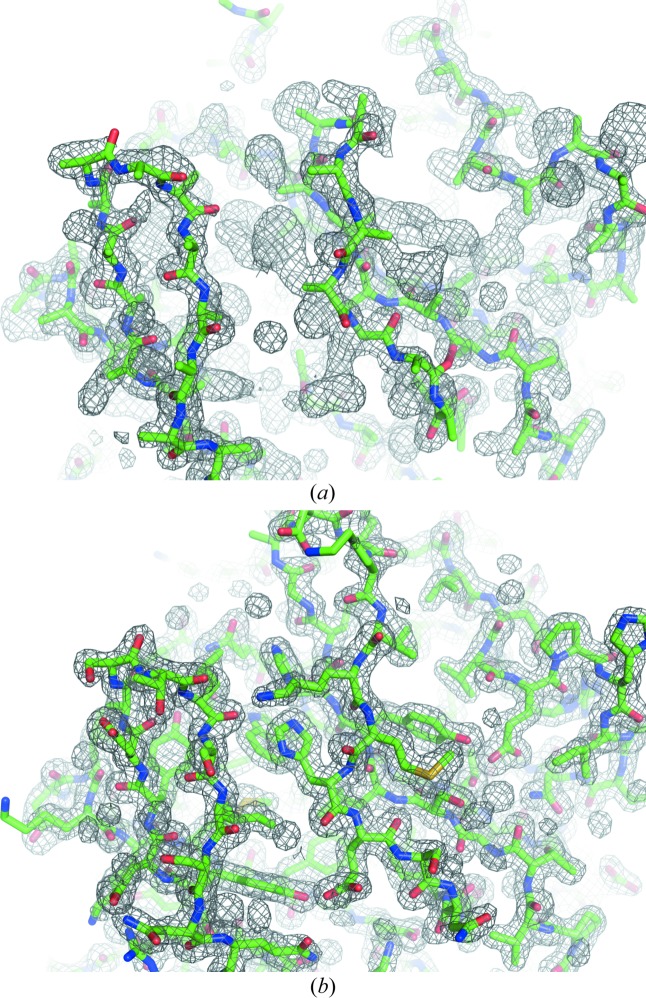
Initial and final maps and models of LRE-Hg. (*a*) An experimentally phased map and traced polyalanine model. (*b*) A 2*mF*_o_ − *DF*_c_ map and refined model. A total of 11 000 indexed patterns of Hg-derivative crystals were used for the calculation. Electron-density maps are contoured at 1.0σ.

We tested various numbers of patterns in the SAD phasing in the same way and found that at least 11 000 patterns (a mean multiplicity of 46.5 at 1.5 Å resolution) were required for SAD phase determination (Fig. 1 ▸). The localization of the Hg site required 11 000 patterns (Fig. S4 in the supporting information). The electron-density map from 11 000 patterns was readily interpretable (Fig. 4 ▸). In the anomalous difference Fourier map, two Hg atoms with peak heights of 30.4σ and 7.1σ were identified near the cysteine residue with occupancies of 0.65 and 0.14, respectively (Fig. S6*c* in the supporting information). The refined structure was consistent with the structure solved by SIRAS phasing (Yamashita *et al.*, 2015 ▸) (root-mean-square deviation of the main-chain atoms of 0.15 Å). Data collection, phasing and refinement statistics for the minimum set of patterns and for all patterns are summarized in Table S4 in the supporting information.

### 3.4. Limits of resolution for successful SAD   

**Figure 5:**
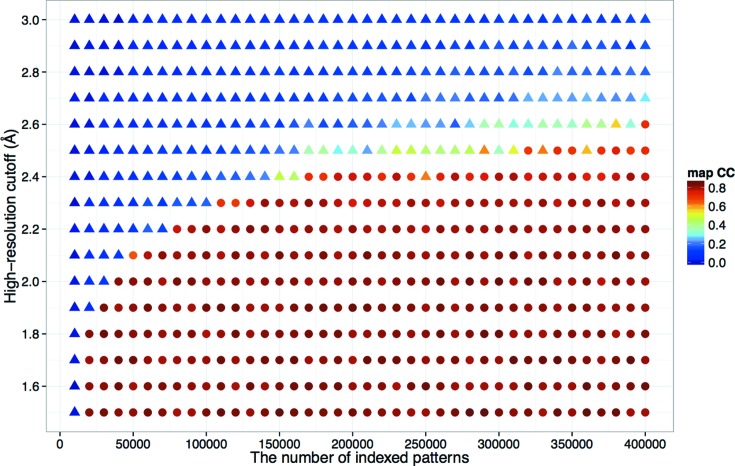
Effect of the high-resolution cutoff and number of patterns in the case of LRE-Hg. The real-space CC of the model built by *Buccaneer* and the final refined 2*mF*_o_ − *DF*_c_ map are indicated by colors, which were calculated using *phenix.get_cc_mtz_pdb* (Adams *et al.*, 2010 ▸). Success (CC 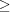 0.65) and failure of phasing are represented as circular and triangular symbols, respectively. This figure was prepared using *ggplot2* (Wickham, 2009 ▸) in *R* (R Development Core Team, 2008 ▸).

In SAD phasing, the phase improvement technique is essential, where high-resolution reflections play an important role, while low-resolution reflections contain a larger anomalous signal that enables substructure determination. To investigate how the high-resolution cutoff affected the phasing, we collected an additional data set for LRE-Hg. Out of 1 268 105 collected images, 542 592 (42.8%) were selected using the *Cheetah* pipeline adapted for SACLA (Nakane, Joti *et al.*, 2016 ▸). Of these, 367 184 (67.7%) patterns were indexed, integrated and merged using the same method as described above. The data set was merged with the original data set described above, and phasing was attempted by varying the number of indexed patterns and the high-resolution cutoff. We found a tendency for a larger number of patterns to enable phasing at lower resolution and SAD phasing was successful at 2.6 Å resolution with 400 000 patterns (a mean multiplicity of 2151.7) (Fig. 5 ▸). For Se-SAD cases, the same trend was observed and phasing at 1.7–2.0 Å was possible with all indexed patterns (Fig. S10 in the supporting information).

### 3.5. Success of SAD phasing and data quality indicators   

In the Monte Carlo integration method, larger numbers of observations yield higher accuracy (Kirian *et al.*, 2010 ▸, 2011 ▸). We investigated the relationship between quality indicators and the success of SAD phasing by changing the number of indexed patterns used for the phasing protocol (statistics by the number of patterns are shown in Fig. 1 ▸; statistics in resolution shells are shown in Fig. S11 in the supporting information).

CC_1/2_ and *R*_split_, two non-anomalous data quality indicators, increased monotonically with the number of patterns. *R*_ano_/*R*_split_ exhibited different behaviors: it increased almost monotonically in the case of Stem-Se and ACG-Se, whereas for LRE-Hg it started to increase at 16 000 patterns. The critical values of *R*_ano_/*R*_split_ for successful SAD phasing were 1.063, 1.035 and 1.053 for Stem-Se, ACG-Se and LRE-Hg, respectively, which are considerably smaller than the value reported for lysozyme-Gd (1.8) (Barends *et al.*, 2014 ▸). This may be attributed to improvements in the phasing programs. 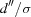 = 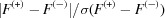 also increased monotonically and SAD phasing was successful at small values (0.876, 0.823 and 0.858 for Stem-Se, ACG-Se and LRE-Hg, respectively). It should be noted that the standard error estimation method used by *CrystFEL* is different from those routinely used in rotation crystallography, which take Poisson noise and systematic errors into account by comparing symmetry-related reflection intensities (Evans, 2011 ▸; Diederichs, 2010 ▸). *CrystFEL* relies on the central limit theorem, which could result in a biased value when SFX multiplicity is low (White *et al.*, 2012 ▸).

CC_ano_ has served as a useful quality indicator for SR experiments. However, a significant CC_ano_ value was not observed here. While the CC_ano_ value was almost zero, the successful SAD phasing reflects sufficient accuracy of the anomalous signal. The low CC_ano_ value of our method likely resulted from the large number of random errors in the measurements compared with the size of the anomalous signal. In the case of lysozyme-Gd (Barends *et al.*, 2014 ▸) CC_ano_ was 0.48, which may have resulted from a much higher Bijvoet ratio (11.9%) compared with the values in our cases (2.2–4.2%). Thus, CC_ano_ may not be a good quality indicator for SAD phasing with SFX data, which has large fluctuations.

In all cases of this study, the anomalous data quality of the low-resolution reflections was limited and hit a peak at ∼2.5 Å for each case (Fig. S11 in the supporting information), unlike conventional crystallography where, generally, reflections at lower resolution have higher data quality. This could be a reason why enormous numbers of patterns were required for SAD phasing of low-resolution data. Further investigation including other SFX cases would be required.

## 4. Conclusion and outlook   

We succeeded in experimental phasing using SeMet-derivatization and Hg-soaking techniques at wavelengths of 0.954 and 0.984 Å, respectively. Such high-energy X-rays, which are essential for Se-SAD, allowed us to collect data at high resolution (1.4–1.6 Å) and perform SAD phasing with 11 000–60 000 indexed patterns.

In this study, we used an MPCCD detector (Kameshima *et al.*, 2014 ▸), which had ∼15–20% (normal to oblique incidence) quantum efficiency (QE) at 13.0 keV as its Si sensor thickness was 50 µm. A detector system with eight MPCCD phase III sensors, which have a sensor thickness of 300 µm and ∼60% QE at 13 keV, is now ready for deployment and expected to enable collection of data with a higher signal-to-noise ratio. In data processing, further improvements by post-refinement and the use of the partiality correction technique may yield phasing-quality data with fewer patterns. There are already a number of post-refinement techniques for XFEL data (White, 2014 ▸; Kabsch, 2014 ▸; Sauter, 2015 ▸; Uervirojnang­koorn *et al.*, 2015 ▸; Ginn *et al.*, 2015 ▸; Kroon-Batenburg *et al.*, 2015 ▸). We are working with H. Ginn to adapt *cppxfel* (Ginn *et al.*, 2016 ▸) for SACLA. Hence, further developments in both hardware and software for SFX will facilitate structural analyses, including *de novo* structure determination, for more challenging targets like membrane proteins and macromolecular complexes.

## 5. Data availability   

The coordinates and experimental data have been deposited in the Protein Data Bank (PDB) with codes 5xfc (Stem-Se 13 000 patterns), 5xfd (ACG-Se 60 000 patterns) and 5xfe (LRE-Hg 11 000 patterns). The raw diffraction images for LRE-Hg are available at CXIDB (http://cxidb.org) with CXIDB ID 31. Those of Stem-Se and ACG-Se have been deposited with CXIDB ID 61 and ID 62, respectively.

## 6. Related literature   

The following references are cited in the Supporting Information for this article: Adams *et al.* (2002 ▸); Grosse-Kunstleve *et al.* (2002 ▸); Karplus & Diederichs (2012 ▸).

## Supplementary Material

Additional figures and tables. DOI: 10.1107/S2052252517008557/lz5015sup1.pdf

PDB reference: Stem-Se, 5xfc

PDB reference: ACG-Se, 5xfd

PDB reference: LRE-Hg, 5xfe

Raw diffraction images for LRE-Hg. URL: https://doi.org/10.11577/1236753

Raw diffraction images for Stem-Se. URL: https://doi.org/10.11577/1365655

Raw diffraction images for ACG-Se. URL: https://doi.org/10.11577/1365656

## Figures and Tables

**Table 1:** The numbers of collected, hit or indexed images and the minimum number required for SAD phasing

Sample	〈|Δ*F*|〉/〈|*F*|〉 (%)†	Space group (*Z*)	No. collected images	No. hit images	No. indexed images	Minimum No. for SAD	Resolution (Å)
Stem-Se	3.7, 4.1	*P*2_1_2_1_2 (4)	91 437	35 295 (38.6%)	26 583 (75.3%)	13 000	1.4
ACG-Se	2.2, 1.4	*P*6_5_ (6)	459 158	163 903 (35.7%)	133 242 (81.3%)	60 000	1.5
LRE-Hg	4.2, 4.5	*P*2_1_2_1_2_1_ (4)	200 079	70 415 (35.2%)	35 235 (50.0%)	11 000	1.5

† The Bijvoet ratio calculated using the approximate equation (Hendrickson & Teeter, 1981 ▸) and the value calculated from *F*_model_ of the refined structure are described. Only the former value can be known before the structure determination.
