# Supplementary material for: Experimental phase determination with selenomethionine or mercury-derivatization in serial femtosecond crystallography
Source: IUCrJ. 2017 Aug 8;4(Pt 5):639–47. doi: 10.1107/S2052252517008557 (PMC5619855; doi:10.1107/S2052252517008557)
Supplement: Supplementary file 1 [file m-04-00639-sup1.pdf]

IUCrJ

Volume 4 (2017)

Supporting information for article:

Experimental phase determination with selenomethionine or mercury-derivatization in serial femtosecond crystallography

Keitaro Yamashita, Naoyuki Kuwabara, Takanori Nakane, Tomohiro Murai, Eiichi Mizohata, Michihiro Sugahara, Dongqing Pan, Tetsuya Masuda, Mamoru Suzuki, Tomomi Sato, Atsushi Kodan, Tomohiro Yamaguchi, Eriko Nango, Tomoyuki Tanaka, Kensuke Tono, Yasumasa Joti, Takashi Kameshima, Takaki Hatsui, Makina Yabashi, Hiroshi Manya, Tamao Endo, Ryuichi Kato, Toshiya Senda, Hiroaki Kato, So Iwata, Hideo Ago, Masaki Yamamoto, Fumiaki Yumoto and Toru Nakatsu

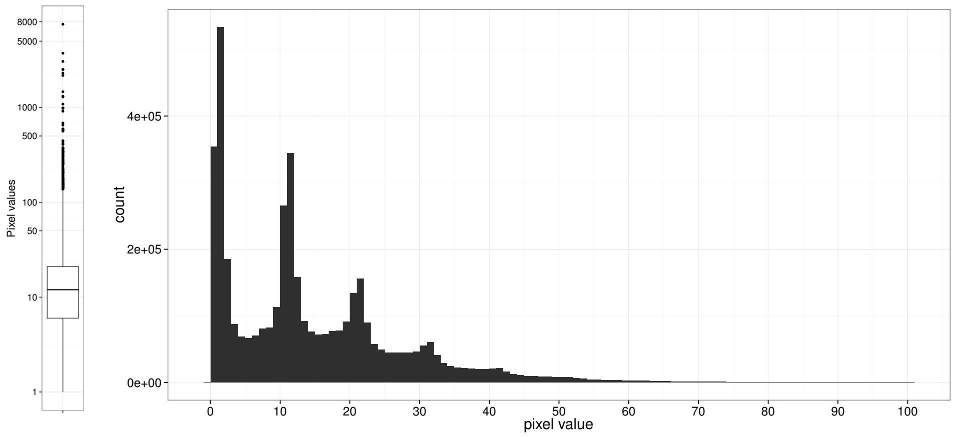

(a) (b)

Fig. S1: **Histogram of detector pixel values.** This figure was prepared using a MPCCD image having a diffraction pattern of LRE-Hg. (a) Whole distribution. (b) Histogram in the range of 0 to 10 photons. Pixel values were scaled so that ten units correspond to one photon by Cheetah [1].

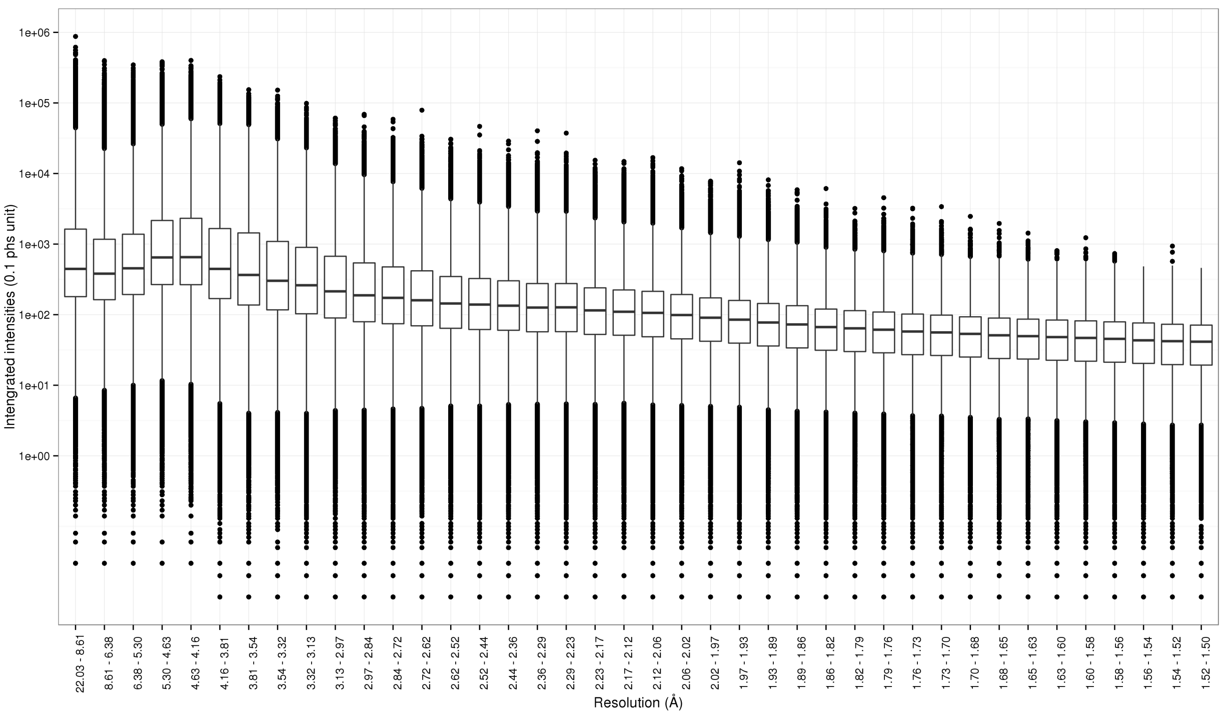
Intengrated intensities (0.1 phs unit)
1e+06
1e+05
1e+04
1e+03
1e+02
1e+01
1e+00
22.03 - 8.61
8.61 - 6.38
6.38 - 5.30
5.30 - 4.63
4.63 - 4.16
4.16 - 3.81
3.81 - 3.54
3.54 - 3.32
3.32 - 3.13
3.13 - 2.97
2.97 - 2.84
2.84 - 2.72
2.72 - 2.62
2.62 - 2.52
2.52 - 2.44
2.44 - 2.36
2.36 - 2.29
2.29 - 2.23
2.23 - 2.17
2.17 - 2.12
2.12 - 2.06
2.06 - 2.02
2.02 - 1.97
1.97 - 1.93
1.93 - 1.89
1.89 - 1.86
1.86 - 1.82
1.82 - 1.79
1.79 - 1.76
1.76 - 1.73
1.73 - 1.70
1.70 - 1.68
1.68 - 1.65
1.65 - 1.63
1.63 - 1.60
1.60 - 1.58
1.58 - 1.56
1.56 - 1.54
1.54 - 1.52
1.52 - 1.50
Resolution (Å)

(a)

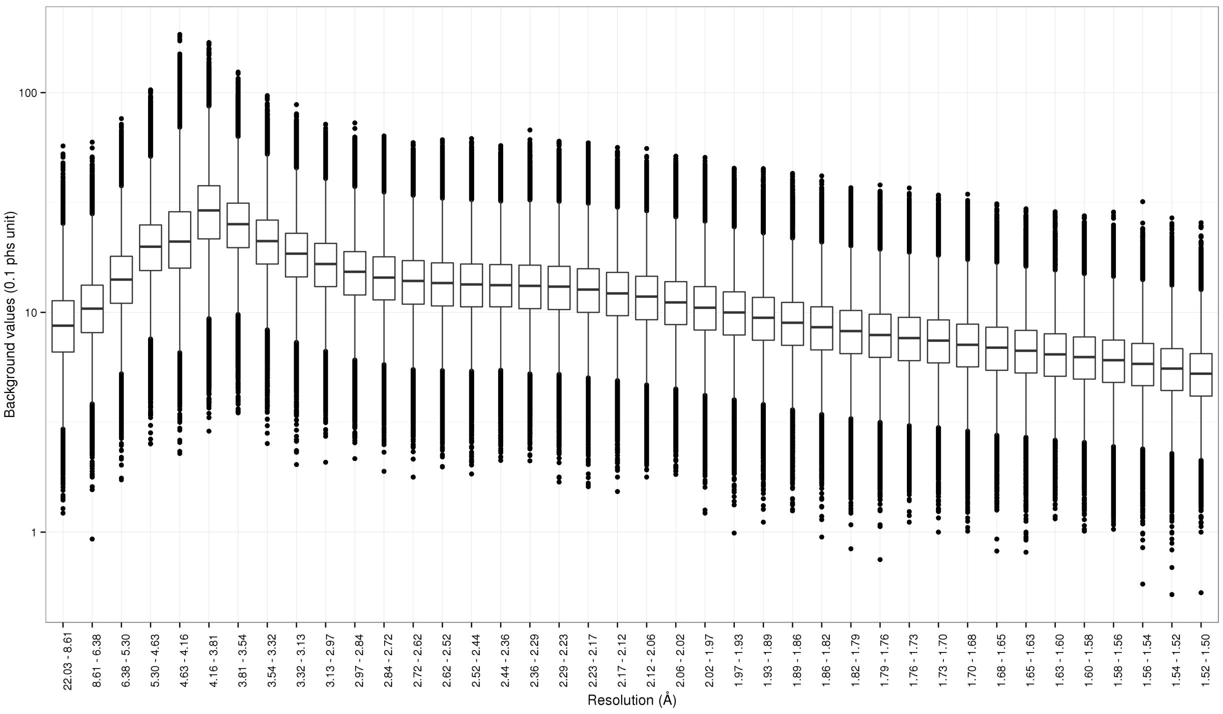
Background values (0.1 phs unit)
100
10
1
22.03 - 8.61
8.61 - 6.38
6.38 - 5.30
5.30 - 4.63
4.63 - 4.16
4.16 - 3.81
3.81 - 3.54
3.54 - 3.32
3.32 - 3.13
3.13 - 2.97
2.97 - 2.84
2.84 - 2.72
2.72 - 2.62
2.62 - 2.52
2.52 - 2.44
2.44 - 2.36
2.36 - 2.29
2.29 - 2.23
2.23 - 2.17
2.17 - 2.12
2.12 - 2.06
2.06 - 2.02
2.02 - 1.97
1.97 - 1.93
1.93 - 1.89
1.89 - 1.86
1.86 - 1.82
1.82 - 1.79
1.79 - 1.76
1.76 - 1.73
1.73 - 1.70
1.70 - 1.68
1.68 - 1.65
1.65 - 1.63
1.63 - 1.60
1.60 - 1.58
1.58 - 1.56
1.56 - 1.54
1.54 - 1.52
1.52 - 1.50
Resolution (Å)

(b)

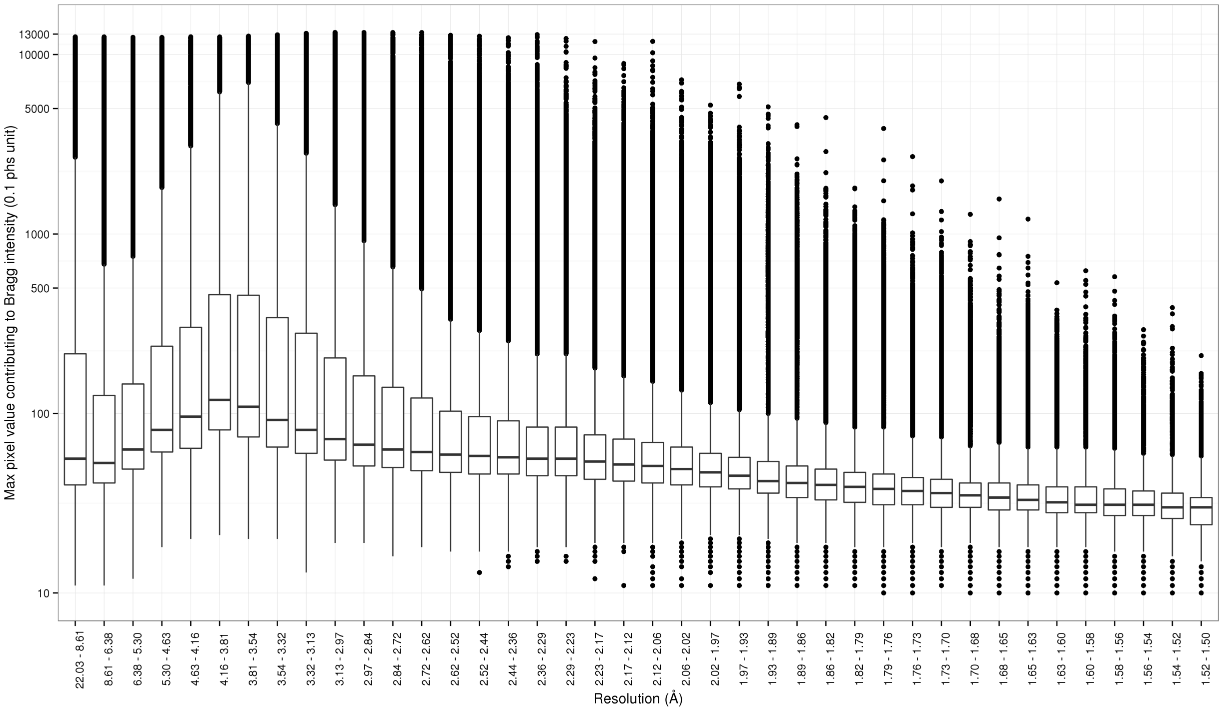

(c)

Fig. S2: **Typical detector pixel values for background and Bragg spots plotted with 13,000 patterns of LRE-Hg.** (a) Integrated intensity. (b) Background. (c) Maximum pixel values contributing to Bragg intensities. The pixel values were scaled so that ten units correspond to one photon by Cheetah [1]. Note that low-angle absorber masked reflections $>\sim 3.8$ Å and corrected values were shown for (a) and (b).

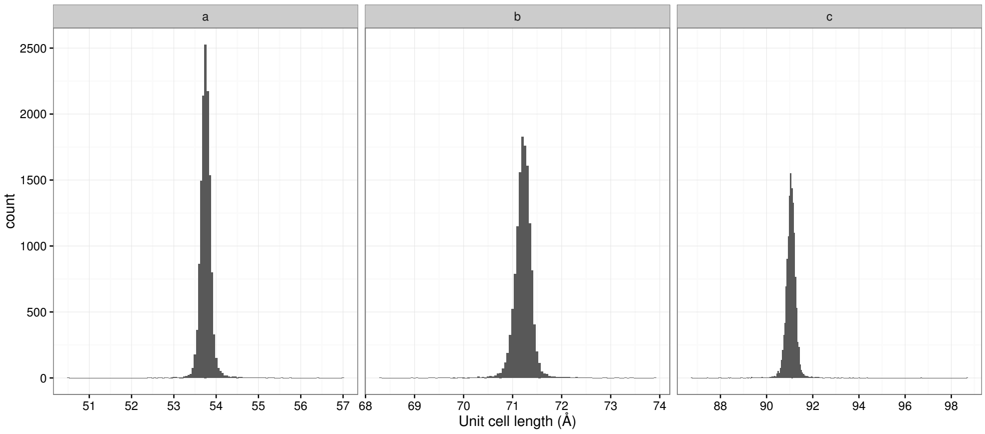

(a)

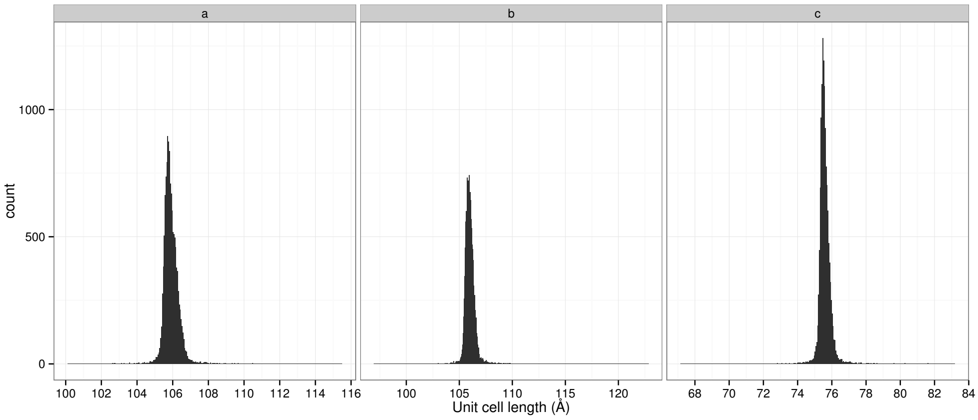

(b)

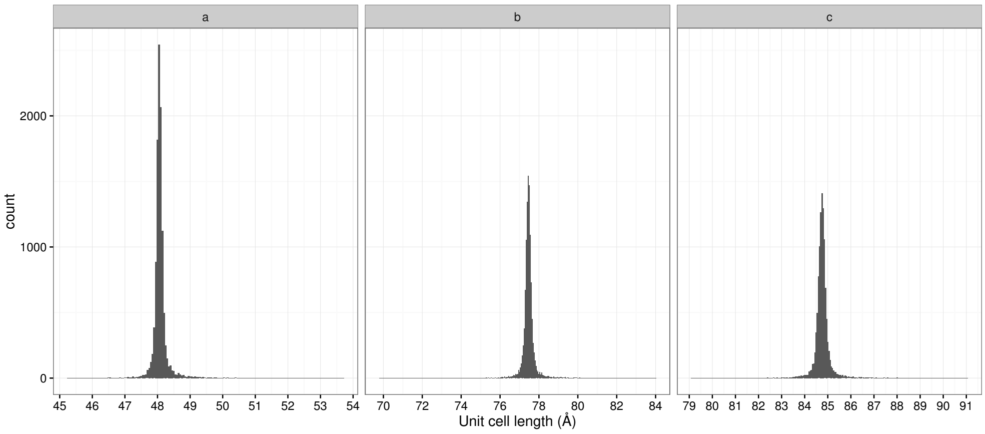

(c)

Fig. S3: **Distribution of unit cell lengths.** (a) 13,000 lattices of Stem, (b) 60,000 lattices of ACG, and (c) 11,000 lattices of LRE. Note that while crystallographically $a = b$ in ACG case, CrystFEL does not take this constraint into account in refinement.

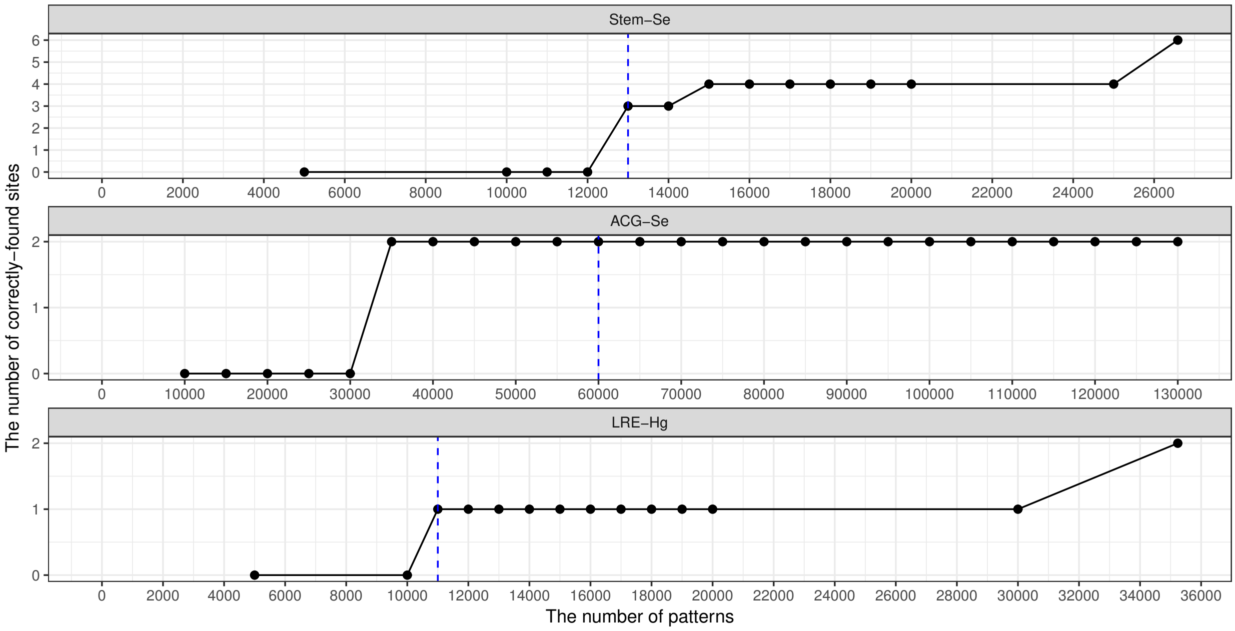

(a)

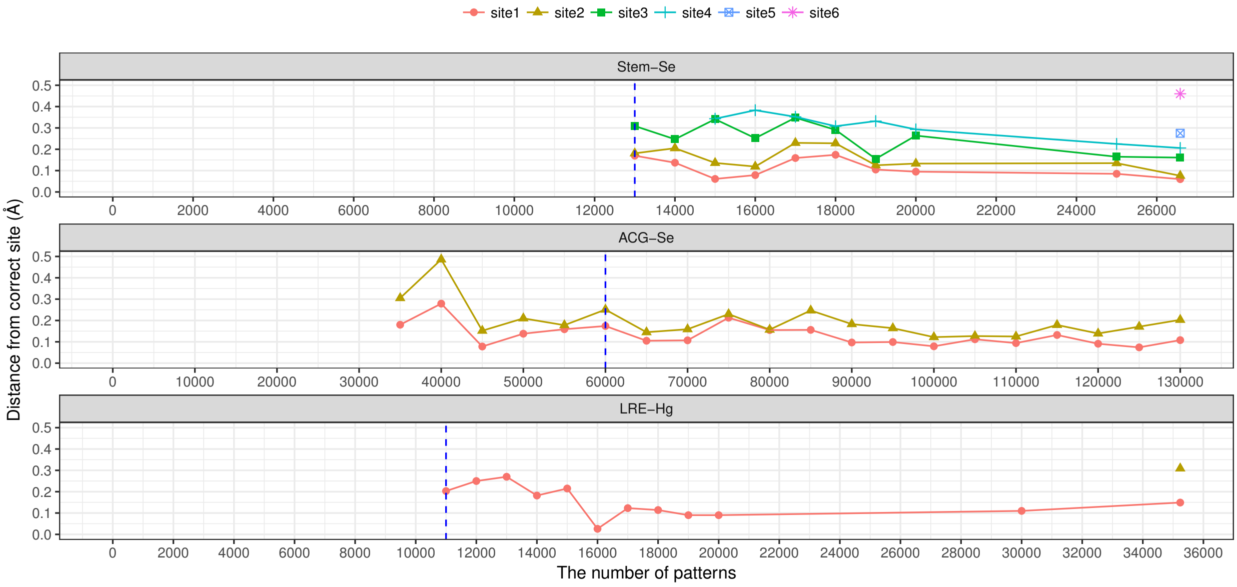

(b)

Fig. S4: **Correctness of heavy atom sites located by SHELXD.** (a) The number of correctly found heavy atom sites. (b) The distances from correct sites. The vertical dashed lines indicate the number of patterns required for successful SAD phasing. Site accuracy was evaluated with phenix.emma [2] by comparing sites in the refined model with the tolerance for matching of 0.5 Å.

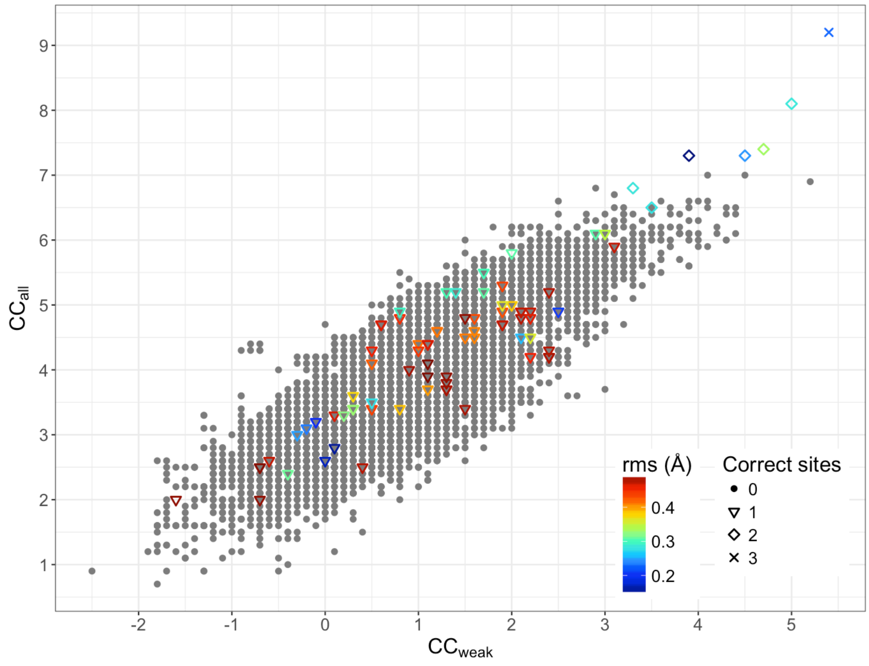

(a)

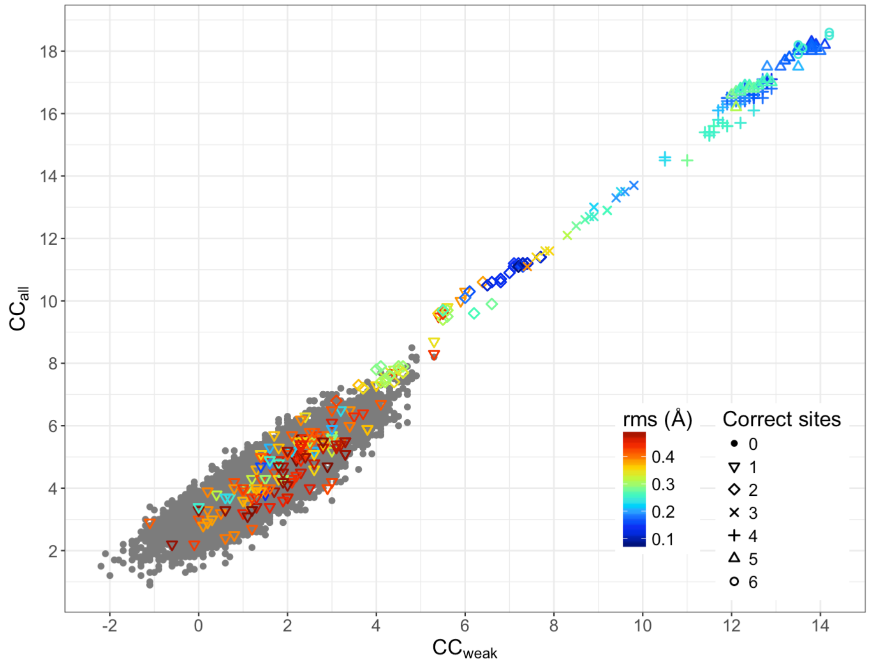

(b)

Fig. S5: **Distribution of CC_{all} and CC_{weak} in SHELXD** with (a) 13,000 patterns and (b) all (26,584) patterns of Stem-Se. Site accuracy was evaluated with phenix.emma [2] by comparing Se sites in refined model with the tolerance for matching of 0.5 Å.

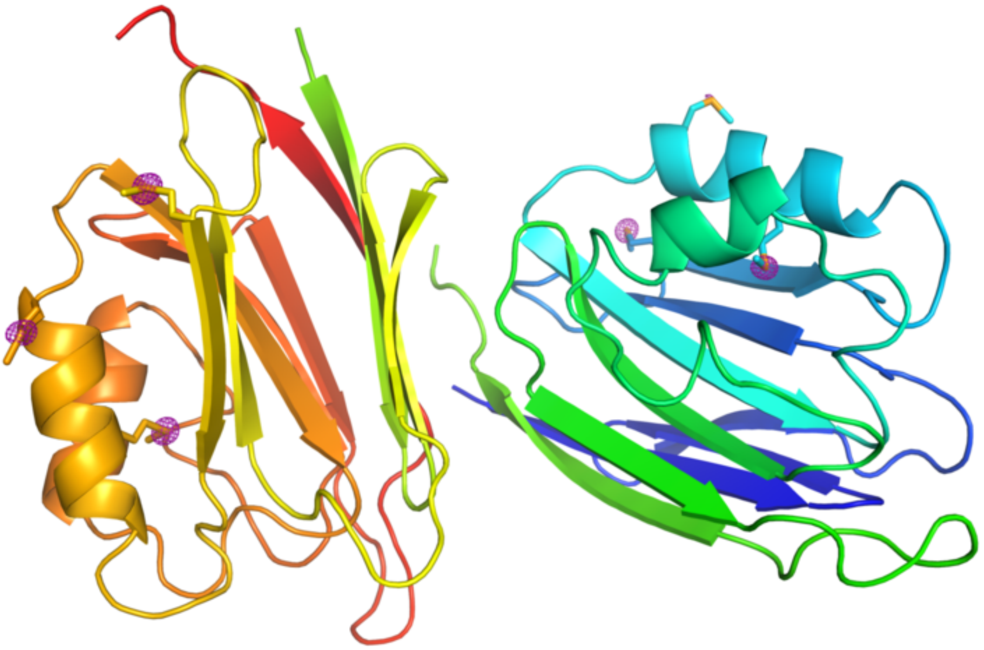

(a)

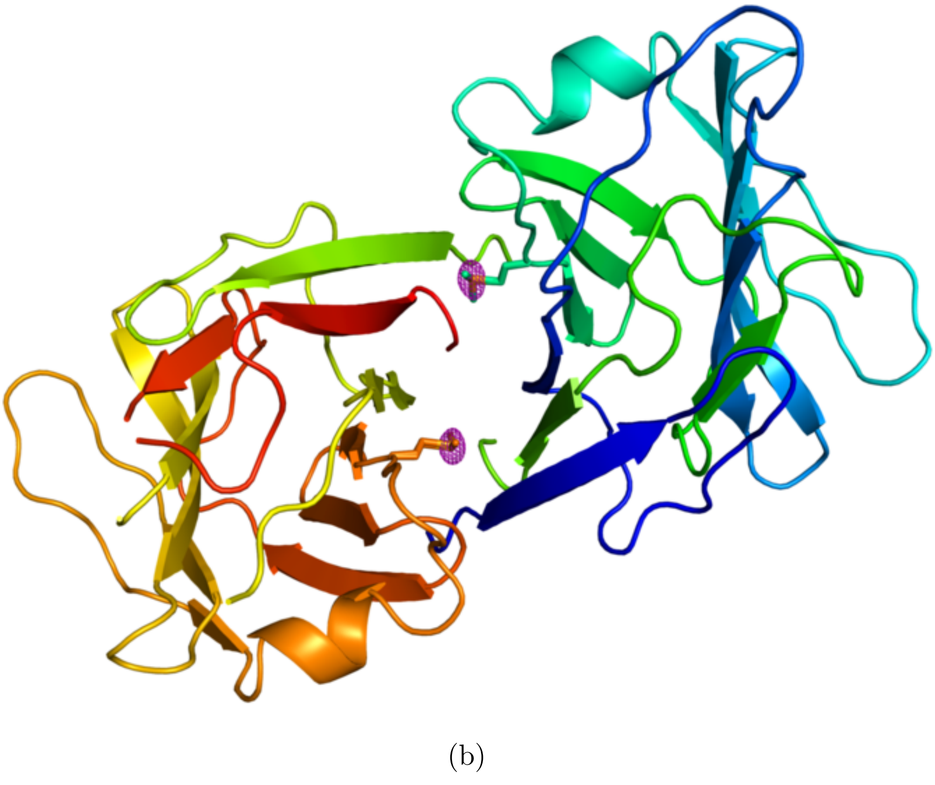

(b)

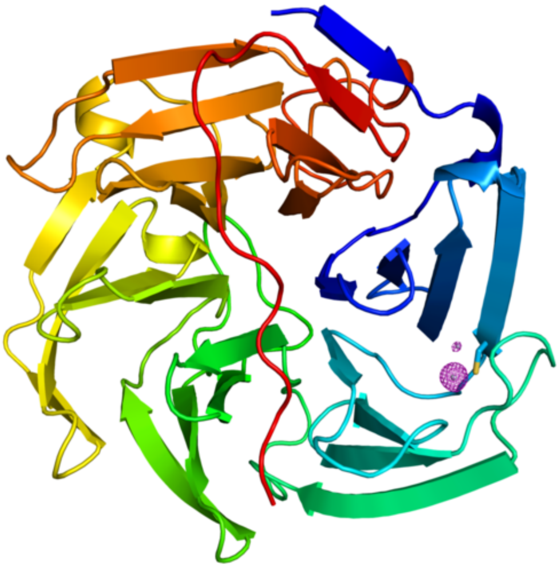

(c)

Fig. S6: **Refined models and anomalous difference Fourier maps of (a) Stem, (b) ACG, (c) LRE.** Anomalous difference Fourier maps are contoured at 6.0σ.

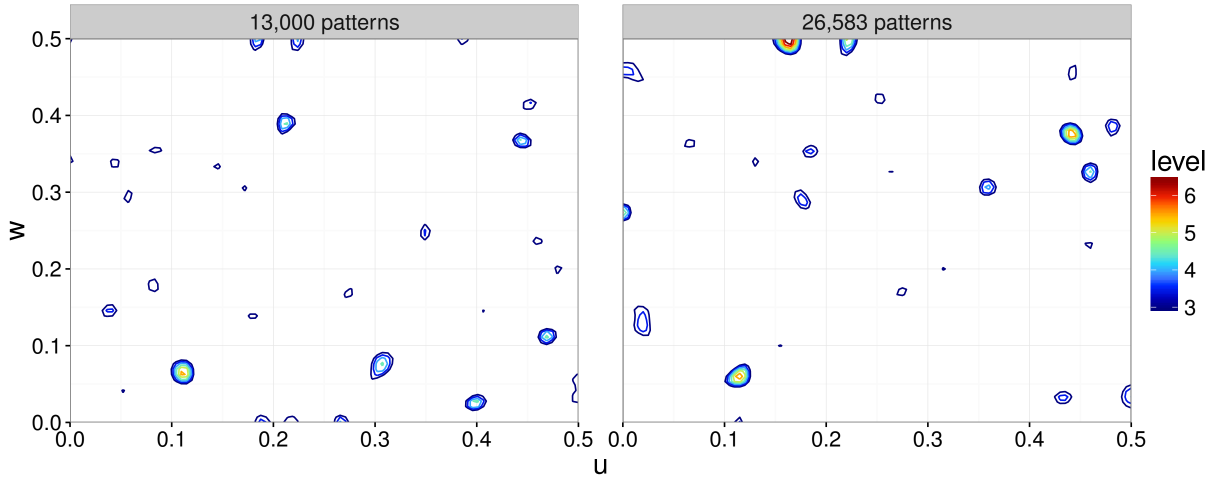

Fig. S7: **Anomalous difference Patterson maps of Stem-Se** in $v = 1/2$ Harker section using 13,000 and 26,583 patterns. Patterson map was calculated using the CCTBX functionality [3] with anomalous differences estimated by SHELXC [4]. The map values are scaled using the root mean square value. Figure was prepared using R [5] with ggplot2 package [6].

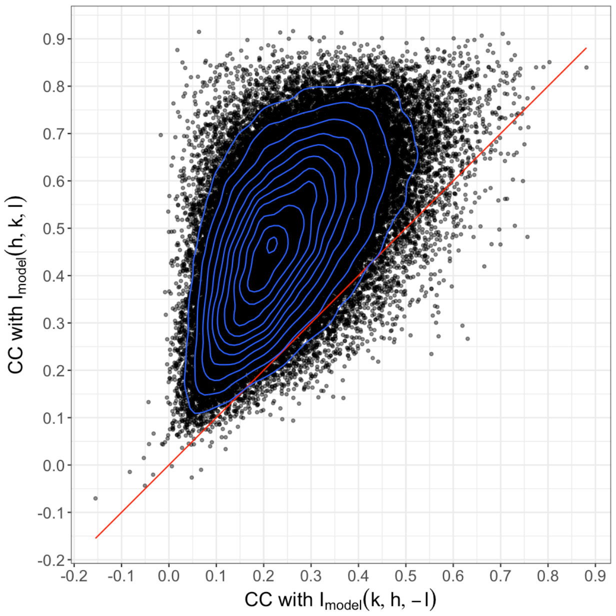

(a)

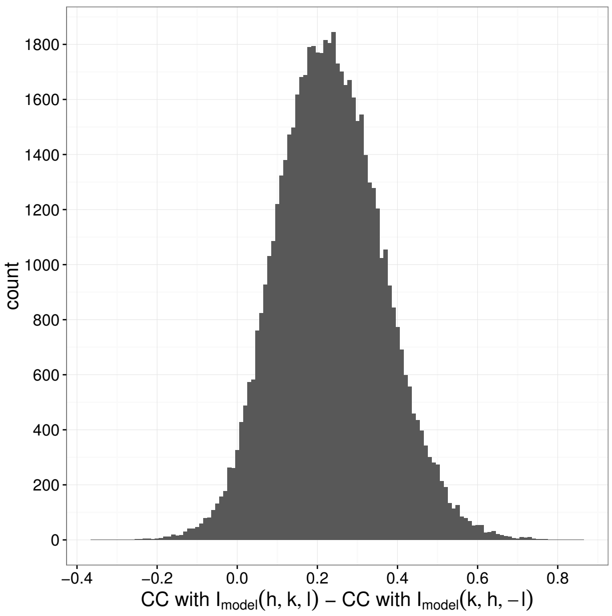

(b)

Fig. S8: **Validation of indexing ambiguity resolution.** Correlation coefficients between measured intensities of each pattern I_{part} and squared calculated structure amplitudes of refined model I_{model} were calculated. Indices of each pattern were reindexed by ambigator. (a) Scatter plot of $\text{CC}[I_{\text{part}}(hkl), I_{\text{model}}(hkl)]$ and $\text{CC}[I_{\text{part}}(hkl), I_{\text{model}}(kh\bar{l})]$, and (b) histogram of $\text{CC}[I_{\text{part}}(hkl), I_{\text{model}}(hkl)] - \text{CC}[I_{\text{part}}(hkl), I_{\text{model}}(kh\bar{l})]$ are shown for the first 60,000 patterns. The result shows indexing ambiguity was correctly resolved for majority of patterns.

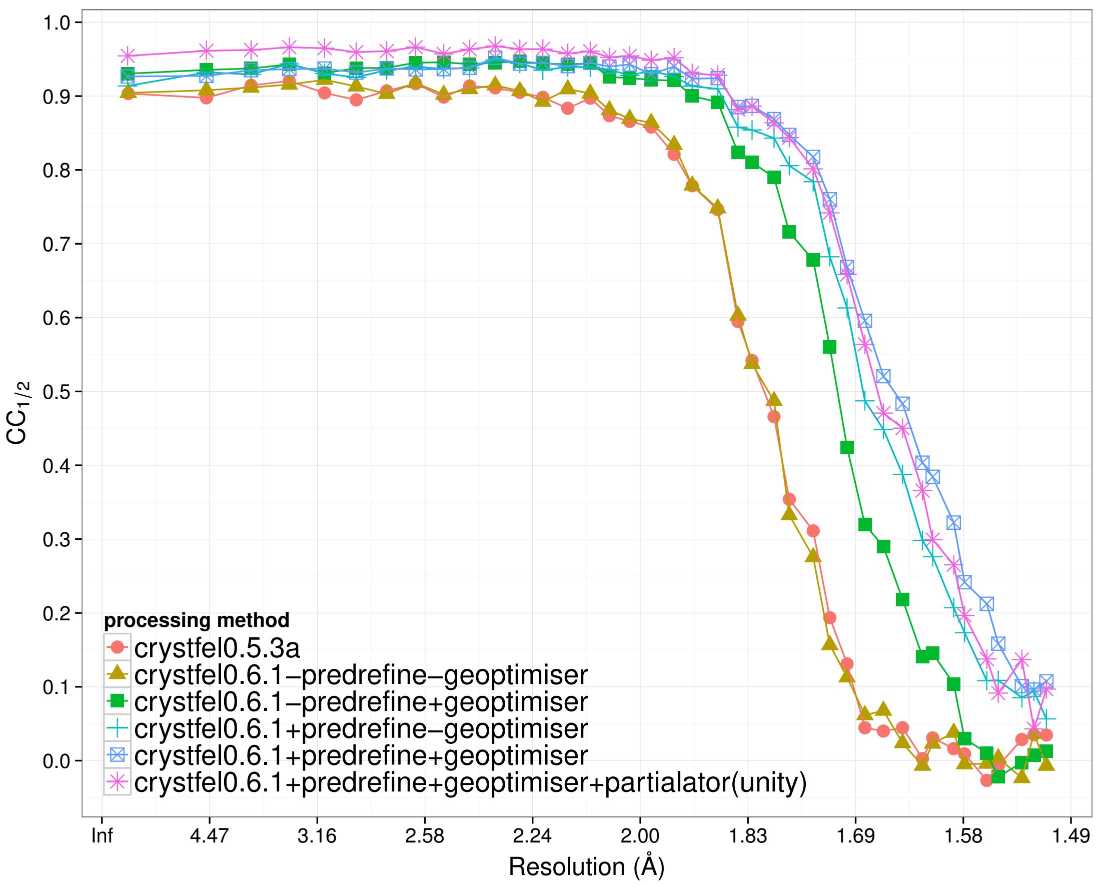

Fig. S9: **Detailed comparison of CrystFEL versions and features using LRE-Hg crystals.** The features of CrystFEL 0.6.1 were compared. 'Predrefine' ('prediction refinement') refines the crystal orientation matrix and detector shifts for each image. Without prediction refinement and geoptimiser, the result was comparable with those obtained using CrystFEL 0.5.3a. The most notable improvement was attributed to prediction refinement, and additional improvement was achieved with geoptimiser. In particular, partialator improved low-resolution data quality. The numbers of indexed patterns were 34,393 (CrystFEL0.5.3a), 35,951 (CrystFEL0.6.1–predrefine–geoptimiser), 40,170 (CrystFEL0.6.1–predrefine+geoptimiser), 34,219 (CrystFEL0.6.1+predrefine–geoptimiser), 35,235 (CrystFEL0.6.1+predrefine+geoptimiser), and 35,231 (CrystFEL0.6.1+predrefine+geoptimiser+partialator). The figure was prepared in R [5] with the ggplot2 package [6].

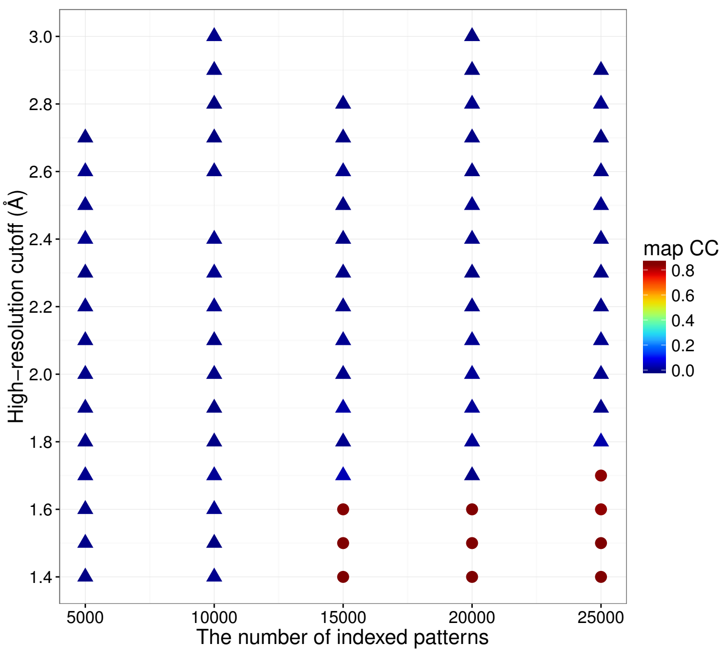

(a)

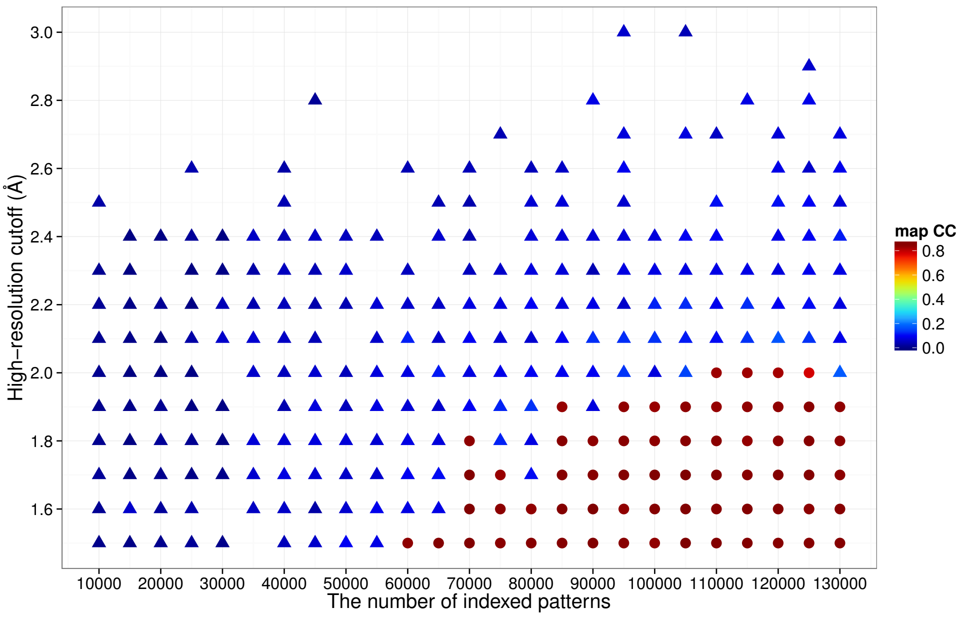

(b)

Fig. S10: **Effect of the high-resolution cutoff and number of patterns in (a) Stem and (b) ACG case.** For LRE-Hg case, see Fig. 5 in the main text.

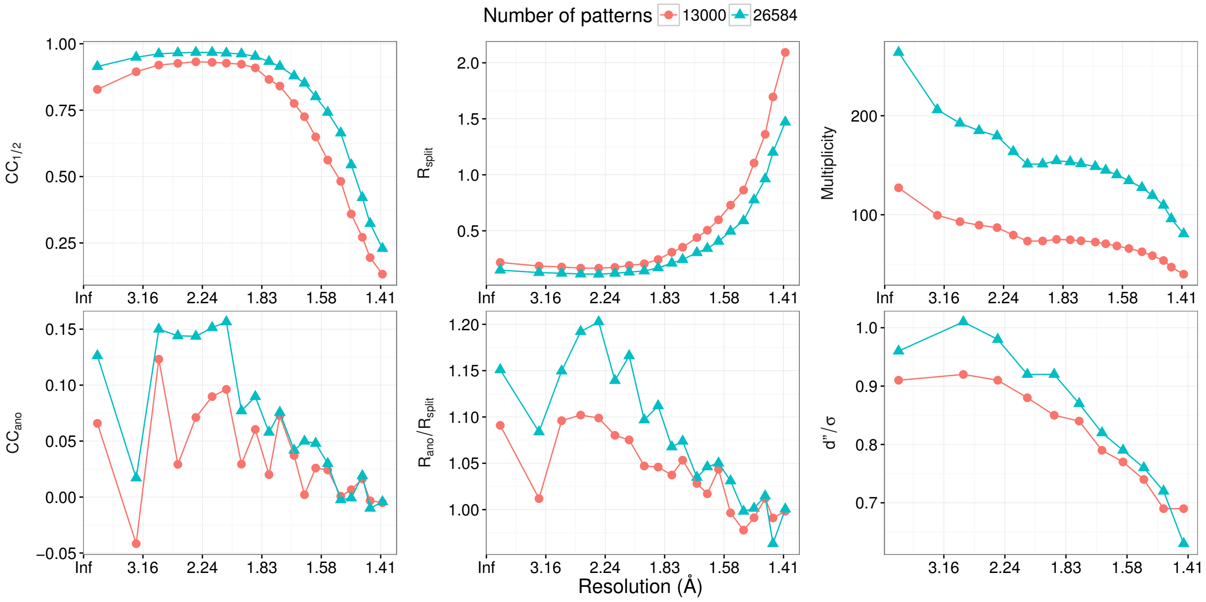
Number of patterns
13000
26584
CC1/2
1.00
0.75
0.50
0.25
Rsplit
2.0
1.5
1.0
0.5
Multiplicity
200
100
CCano
0.15
0.10
0.05
0.00
−0.05
Rano/Rsplit
1.20
1.15
1.10
1.05
1.00
d''/σ
1.0
0.9
0.8
0.7
Inf
3.16
2.24
1.83
1.58
1.41
Resolution (Å)

(a)

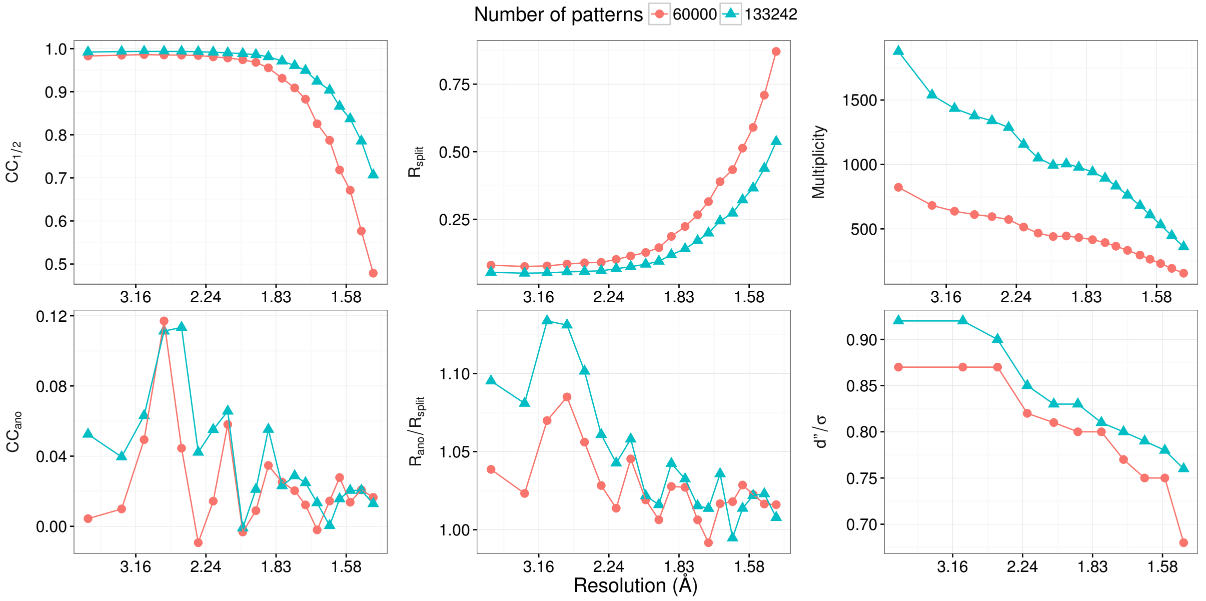
Number of patterns
60000
133242
CC1/2
1.0
0.9
0.8
0.7
0.6
0.5
Rsplit
0.75
0.50
0.25
Multiplicity
1500
1000
500
CCano
0.12
0.08
0.04
0.00
Rano/Rsplit
1.10
1.05
1.00
d''/σ
0.90
0.85
0.80
0.75
0.70
3.16
2.24
1.83
1.58
Resolution (Å)

(b)

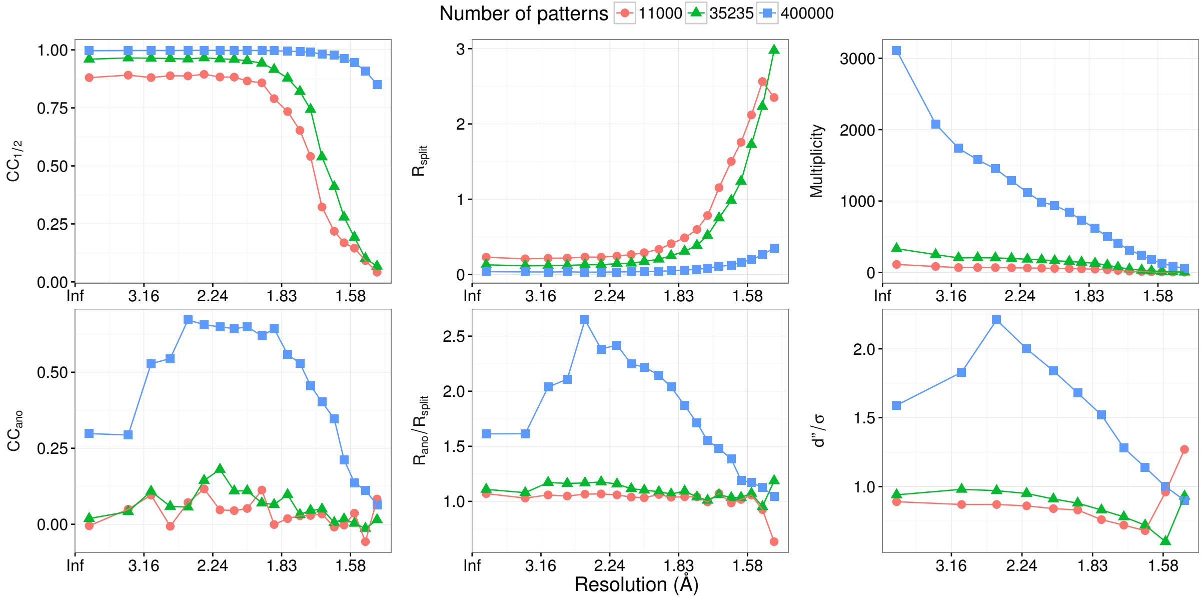

(c)

Fig. S11: **Data processing statistics in resolution shells.** $CC_{1/2}$, R_{split}, multiplicity, CC_{ano}, $R_{\mathrm{ano}}/R_{\mathrm{split}}$, and d''/σ are shown. (a) Stem. (b) ACG. (c) LRE.

Table S1: XFEL experimental parameters

	LRE-Hg	LRE-Hg (additional)	Stem-Se	ACG-Se
Carrier	Super Lube (#21030, Synco Chemical Co.)	Super Lube (#21030, Synco Chemical Co.)	Super Lube (#21030, Synco Chemical Co.)	Super Lube Nuclear grease (#42150, Synco Chemical Co.)
Crystal size (μm) and shape	2–5×10–30, rod shape	2–5×10–30, rod shape	10–30, cube-like	5–50×10–60, hexagonal prism shape
Filter pore size (μm)	30	30	30	30
Crystal density in stream (/mL)	2×10^7	7.8×10^7	1.6×10^7	1.2×10^7
Nozzle inner diameter (μm)	110	110	160	150
Flow rate (μL/min, μm/s)	0.48–0.50 / 876	0.25 / 438	0.50 / 417	0.625 / 590
Beam size (width×height /μm)	2.1 × 2	2.3 × 1.4	1.9 × 1.21	1.4 × 1.2
Photon energy (keV)	12.6	12.6	13.0	13.0
Energy bandwidth (FWHM, eV)	n/a	44	92	51
Attenuators (transmittance)	Al 300 μm +/- Si 100 μm (36.4% or 24.9%)	None, Al 25 μm, or Al 50 μm (91.9%, 84.5%)	None	None or Al 50 μm (85.8%)
Averaged beam energy† (μJ/pulse)	86.4	97.6	192.8	296
Detector distance (edge resolution)	81.4–82.2 mm (1.7 Å)	55.5 mm (1.36 Å)	54.3 mm (1.30 Å)	55 mm (1.32 Å)
Low-angle absorber (Al thickness /μm, boundary resolution /Å)	300 / 3.8	300 / 2.7	Not used	Not used

†XFEL beam energy at sample position calculated from the reflectivity or transmittance of the components between the beam monitor and the sample position (attenuator, KB-mirrors, Be windows, and air path).

Table S2: Data collection and refinement statistics of Stem-SeMet derivative

	minimum patterns	all patterns
Space group	$P2_12_12$	$P2_12_12$
Unit cell (a, b, c; Å)	71.2, 91.1, 53.8	71.2, 91.1, 53.8
Resolution range† (Å)	24.0–1.40 (1.46–1.40)	24.0–1.40 (1.46–1.40)
Completeness† (%)	100 (100)	100 (100)
SFX multiplicity†	74.3 (44.3)	152.7 (89.9)
No. crystals	13,000	26,583
$R_{\text{split}}{}^{\dagger\S}$	0.2115 (1.838)	0.1451 (1.302)
$\langle I/\sigma(I)\rangle$	3.3 (0.63)	4.7 (0.87)
$\text{CC}_{1/2}{}^{\dagger\#}$	0.9321 (0.1727)	0.9679 (0.2877)
$\text{CC}_{\text{ano}}{}^{*}$	0.03967	0.09875
**SAD phasing**		
CC_{all}, CC_{weak} (%; SHELXD)	9.21, 5.42	18.55, 14.19
Built residues, CC, FOM (SHELXE)	285, 42.5%, 0.649	291, 46.68%, 0.688
Rebuilt residues, $R_{\text{work}}/R_{\text{free}}$ (Buccaneer)	300, 24.2%/31.6%	307, 25.2%/30.3%
**Refinement**		
$R_{\text{work}}, R_{\text{free}}$	0.1704, 0.2067	0.1609, 0.1901
No. atoms (mean B-factor)		
protein	2346 (25.1)	2346 (24.9)
water	171 (39.0)	171 (38.6)
ligand	42 (27.1)	42 (26.7)
r.m.s. deviation from ideal		
bond lengths (Å)	0.005	0.005
bond angles (°)	1.026	1.033
Ramachandran plot		
Favored (%)	98.68	98.68
Allowed (%)	1.32	1.32
Outlier (%)	0	0

†Values in parenthesis are for the highest-resolution shell.

$^\S R_{\text{split}} = \frac{1}{\sqrt{2}} \frac{\sum_{hkl} |I_{hkl}^{\text{even\_images}} - I_{hkl}^{\text{odd\_images}}|}{\sum_{hkl}(I_{hkl}^{\text{even\_images}} + I_{hkl}^{\text{odd\_images}})/2}$ [7], $^\#\text{CC}_{1/2}$ is a correlation coefficient of intensities between randomly halved datasets [8]. $^*\text{CC}_{\text{ano}}$ is a correlation coefficient of anomalous intensity differences between randomly halved datasets.

Table S3: Data collection and refinement statistics of ACG-SeMet derivative

	minimum patterns	all patterns
Space group	$P6_5$	$P6_5$
Unit cell ($a = b, c$; Å)	105.8, 75.5	105.8, 75.5
Resolution range† (Å)	9.5–1.50 (1.56–1.50)	9.5–1.50 (1.56–1.50)
Completeness† (%)	100 (100)	100 (100)
SFX multiplicity†	443.4 (179.0)	1004.5 (413.2)
No. crystals	60,000	133,242
R_{split}	0.0958 (0.768)	0.0633 (0.475)
$\langle I/\sigma(I)\rangle$	6.46 (1.45)	9.84 (2.32)
$CC_{1/2}$	0.9903 (0.5421)	0.9956 (0.7576)
CC_{ano}	0.0105	0.0511
**SAD phasing**		
CC_{all}, CC_{weak} (%; SHELXD)	10.72, 5.54	16.01, 9.42
Built residues, CC, FOM (SHELXE)	192, 23.55%, 0.741	206, 27.76%, 0.775
Rebuilt residues, $R_{\text{work}}/R_{\text{free}}$ (Buccaneer)	318, 24.0%/24.9%	324, 23.4%/24.2%
**Refinement**		
$R_{\text{work}}, R_{\text{free}}$	0.1488, 0.1625	0.1414, 0.1534
No. atoms (mean B-factor)		
protein	2432 (22.7)	2444 (22.1)
water	232 (40.2)	224 (38.9)
ligand	100 (22.6)	100 (21.5)
r.m.s. deviation from ideal		
bond lengths (Å)	0.019	0.019
bond angles (°)	1.478	1.459
Ramachandran plot		
Favored (%)	97.19	96.57
Allowed (%)	2.81	3.43
Outlier (%)	0	0

†Values in parenthesis are for the highest-resolution shell.

Table S4: Data collection and refinement statistics of LRE-Hg derivative

	minimum patterns	all patterns
Space group	$P2_12_12_1$	$P2_12_12_1$
Unit cell (a, b, c; Å)	47.9, 77.0, 84.5	47.9, 77.0, 84.5
Resolution range† (Å)	25–1.50 (1.56–1.50)	25–1.50 (1.56–1.50)
Completeness† (%)	96.73 (71.46)	99.19 (92.74)
SFX multiplicity†	46.5 (3.6)	132.4 (6.1)
No. crystals	11,000	35,231
R_{split}	0.2476 (2.598)	0.1474 (2.344)
$\langle I/\sigma(I)\rangle$	2.7 (0.48)	4.4 (0.63)
$CC_{1/2}$	0.9245 (0.07867)	0.9755 (0.1006)
CC_{ano}	0.01169	0.02952
**SAD phasing**		
CC_{all}, CC_{weak} (%; SHELXD)	4.87, 2.39	15.71, 9.85
Built residues, CC, FOM (SHELXE)	292, 37.66%, 0.599	293, 41.87%, 0.635
Rebuilt residues, $R_{\text{work}}/R_{\text{free}}$ (Buccaneer)	308, 25.4%/26.7%	309, 23.5%/25.3%
**Refinement**		
$R_{\text{work}}, R_{\text{free}}$	0.1916, 0.2134	0.1755, 0.1976
No. atoms (mean B-factor)		
protein	2397 (24.4)	2397 (24.2)
water	162 (34.3)	162 (34.1)
ligand/ion	9 (27.2)	9 (27.0)
Hg	2 (19.5)	2 (19.3)
r.m.s. deviation from ideal		
bond lengths (Å)	0.007	0.007
bond angles (°)	1.130	1.129
Ramachandran plot		
Favored (%)	95.75	95.75
Allowed (%)	4.25	4.25
Outlier (%)	0	0

†Values in parenthesis are for the highest-resolution shell.

[1] Nakane, T. *et al.* Data processing pipeline for serial femtosecond crystallography at SACLA. *Journal of Applied Crystallography* **49**, 1035–1041 (2016).

[2] Adams, P. D. *et al. PHENIX*: building new software for automated crystallographic structure determination. *Acta Crystallographica Section D* **58**, 1948–1954 (2002).

[3] Grosse-Kunstleve, R. W., Sauter, N. K., Moriarty, N. W. & Adams, P. D. The *Computational Crystallography Toolbox*: crystallographic algorithms in a reusable software framework. *Journal of Applied Crystallography* **35**, 126–136 (2002).

[4] Sheldrick, G. M. Experimental phasing with *SHELXC/D/E*: combining chain tracing with density modification. *Acta Crystallographica Section D* **66**, 479–485 (2010).

[5] R Development Core Team. *R: A Language and Environment for Statistical Computing.* R Foundation for Statistical Computing, Vienna, Austria (2008).

[6] Wickham, H. *ggplot2: elegant graphics for data analysis* (Springer New York, 2009).

[7] White, T. A. *et al. CrystFEL*: a software suite for snapshot serial crystallography. *Journal of Applied Crystallography* **45**, 335–341 (2012).

[8] Karplus, P. A. & Diederichs, K. Linking Crystallographic Model and Data Quality. *Science* **336**, 1030–1033 (2012).
